# DNA is loaded through the 9-1-1 DNA checkpoint clamp in the opposite direction of the PCNA clamp

**DOI:** 10.1038/s41594-022-00742-6

**Published:** 2022-03-21

**Authors:** Fengwei Zheng, Roxana E. Georgescu, Nina Y. Yao, Michael E. O’Donnell, Huilin Li

**Affiliations:** 1 Department of Structural Biology, Van Andel Institute, Grand Rapids, MI USA; 2 DNA Replication Laboratory, The Rockefeller University, New York, NY USA; 3 Howard Hughes Medical Institute, The Rockefeller University, New York, NY USA

**Keywords:** Cryoelectron microscopy, DNA metabolism

## Abstract

The 9-1-1 DNA checkpoint clamp is loaded onto 5′-recessed DNA to activate the DNA damage checkpoint that arrests the cell cycle. The 9-1-1 clamp is a heterotrimeric ring that is loaded in *Saccharomyces cerevisiae* by Rad24-RFC (hRAD17-RFC), an alternate clamp loader in which Rad24 replaces Rfc1 in the RFC1-5 clamp loader of proliferating cell nuclear antigen (PCNA). The 9-1-1 clamp loading mechanism has been a mystery, because, unlike RFC, which loads PCNA onto a 3′-recessed junction, Rad24-RFC loads the 9-1-1 ring onto a 5′-recessed DNA junction. Here we report two cryo-EM structures of Rad24-RFC–DNA with a closed or 27-Å open 9-1-1 clamp. The structures reveal a completely unexpected mechanism by which a clamp can be loaded onto DNA. Unlike RFC, which encircles DNA, Rad24 binds 5′-DNA on its surface, not inside the loader, and threads the 3′ ssDNA overhang into the 9-1-1 clamp from above the ring.

## Abstract (web summary)

Cryo-EM analysis reveals that the 9-1-1 clamp loader Rad24-RFC recognizes the 5′-recessed DNA end. The Rad24 subunit holds the DNA above the clamp, thereby loading the 9-1-1 clamp in the opposite direction of RFC loading DNA into the PCNA clamp.

## Main

**Fig. 1:**
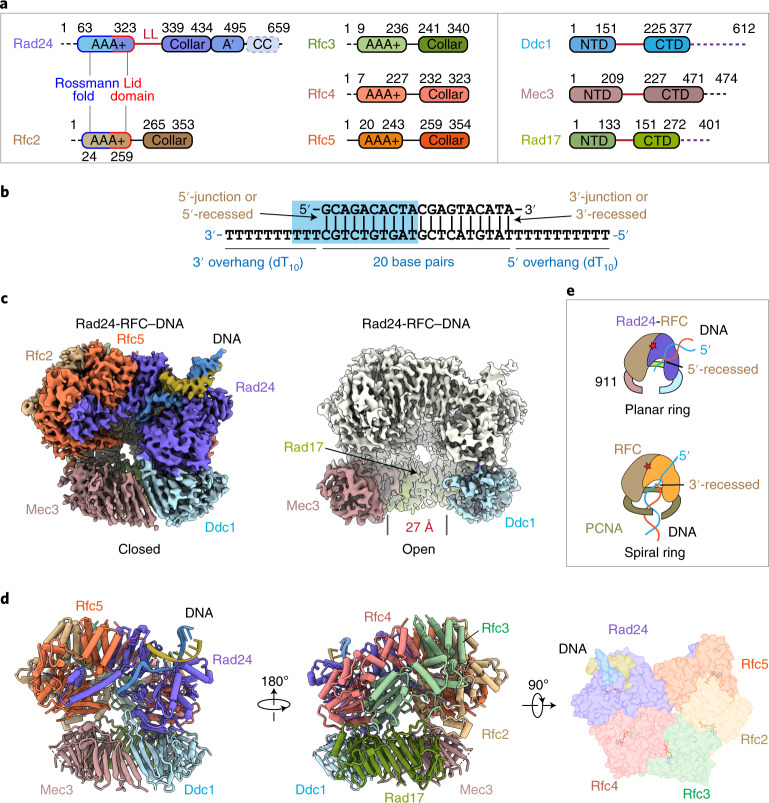
Cryo-EM 3D maps of the *S.cerevisiae* Rad24-RFC–9-1-1–DNA ternary complex. **a**, Domain architecture of the eight proteins of the Rad24-RFC–9-1-1 complex: the clamp loader proteins Rad24 and Rfc2–5 and the 9-1-1 clamp proteins Ddc1, Mec3 and Rad17. The C-terminal collar domain forms the tight pentamer connections. The Rad24 A′ domain is associated with the collar domain that is also present in Rfc1, which Rad24 replaces. The unique Rad24 C-terminal coiled coil (CC) is disordered. The dashed lines indicate unsolved regions. LL is the long linker between the Rad24 AAA+ and collar domains. The red lines between the NTDs and CTDs of the 9-1-1 subunits represent the IDCL between the two domains of each 9-1-1 subunit, and the dashed purple lines represent the long C-terminal tails of Ddc1 and Rad17. **b**, The DNA substrate used to form the ternary complex. Both a 5′- and 3′-recessed DNA junction are present. Nucleotides in the cyan box are resolved in the structure. **c**, Segmented 3D maps of the Rad24-RFC–9-1-1 clamp–DNA complex in closed (left) and open (right) states. Maps are surface-rendered at 0.1 threshold, except for Ddc1 in the open conformation, which is separately displayed at 0.07 threshold. **d**, Model of the closed-state complex in front (left) and back (middle) cartoon view and in a top (N-terminal) surface view (right). **e**, Sketch comparing the DNA binding mode of the Rad24-RFC–9-1-1 (upper panel, this study) with that of the RFC–PCNA, based on the T4 clamp–clamp loader–DNA structure (lower panel; PDB 3U60). Note the drastically different DNA positions and the spiral versus planar clamp rings of the two systems.

The DNA damage response is essential for maintaining genome integrity in all eukaryotes^1–4^. In response to DNA damage or replication stress, a checkpoint signaling pathway is activated to arrest the cell cycle and initiate repair of the DNA damage, or trigger apoptosis^5–10^. Following DNA damage, single-stranded (ss) DNA is produced by recombination and repair reactions and by CMG (Cdc45, Mcm2–7, GINS) helicase advance, which continues after replicative DNA polymerases stall at sites of DNA damage. The ssDNA gaps contain 5′ ss/double-stranded (ds) DNA junctions (referred to here as a ‘5′-recessed DNA junction’). Such 5′-recessed DNA junctions provide ‘targets of opportunity’ to signal that DNA damage has occurred. In both yeast and human, the DNA damage response can be initiated by loading of the checkpoint complex, Rad9–Hus1–Rad1 in human or Ddc1–Mec3–Rad17 in yeast (both referred to as 9-1-1; Fig. 1a), onto 5′-recessed DNA junctions produced as a consequence of DNA damage^11–13^. The 9-1-1 clamp loading is accomplished by a specialized clamp loader called RAD17-RFC (RFC, replication factor C) in human and Rad24-RFC in *Saccharomyces cerevisiae*^14^.

There is a rich biochemical background of 9-1-1 clamp loading. The 9-1-1 complex is a proliferating cell nuclear antigen (PCNA)-like ring that is conserved from yeast to human^15–18^. Biochemical studies show that Rad24(hRAD17)-RFC loads the 9-1-1 clamp onto DNA and that the clamp can slide on DNA^11–13,19–21^. Crystal structures have shown that each 9-1-1 subunit has a PCNA-like core consisting of an N-terminal domain (NTD) and a C-terminal domain (CTD) that are connected by a long inter-domain connecting loop (IDCL)^22–24^. The inner chamber of the 9-1-1 ring is lined by basic residues that facilitate DNA binding and/or sliding. The human 9-1-1 subunit RAD9 (yDdc1) has a long C-tail with phosphorylation sites that recruits TopBP1 (yDpb11) to activate the ATR kinase, ataxia telangiectasia (yMec1)^25,26^. The active kinase phosphorylates and activates checkpoint kinase 1 (yRad53)^27,28^, which phosphorylates numerous proteins, leading to arrest of the cell cycle^4,5^.

Despite their similar ring structure, 9-1-1 and PCNA have very different functions. PCNA is a homotrimer sliding clamp for processive replicative DNA polymerases (Pol), and also interacts with many translesion Pols and other DNA metabolic factors^29–33^. The PCNA clamp is loaded onto a 3′-template/primer DNA by the RFC loader^34^. In contrast, the hRAD17(yRad24)-RFC clamp loader loads the 9-1-1 clamp onto a 5′-recessed DNA junction^11,13^.

RAD17(Rad24)-RFC is one of three alternate RFC-like complexes in eukaryotes in which RFC1 is replaced by another subunit^35^. In this report, *Saccharomyces cerevisiae* (*S.c.*) Rad24 replaces Rfc1 and loads the DNA damage 9-1-1 ring onto DNA to signal DNA damage (Fig. 1a). The 9-1-1 clamp loading process is assisted by replication protein A (RPA), which covers the ssDNA adjacent to the 5′-recessed DNA junction^19^.

The structures of the yeast and human RFC–PCNA complexes in the absence of DNA show that each Rfc subunit contains the AAA+ (ATPases associated with diverse cellular activities) module, composed of a Rossmann domain and a helical lid domain^36^, and a C-terminal collar domain^37–39^. The RFC pentamer is configured in a right-hand spiral consisting of two tiers, with five C-terminal collar domains forming a sealed ring at the top tier, and five AAA+ modules forming a spiral with a gap between Rfc1 and Rfc5 at the lower tier^40–42^. Rfc1 and Rad24 have an additional A′ domain that connects with the adjacent Rfc5 AAA+ module. The adenosine triphosphate (ATP) binding sites of the RFC loaders are located at the interfaces of adjacent AAA+ modules, with one AAA+ module binding the ATP via the conserved Walker A (P-loop) and Walker B (DExx box) motifs, and the neighboring AAA+ module contributing an arginine finger within a conserved Ser-Arg-Cys (SRC) motif, and a central helix motif^34,40–42^. ATP hydrolysis requires the ATP site elements of both subunits, but neither Rfc1 nor Rad24 have the SRC motif and central helix motif that are conserved in Rfc2–5—features that are also conserved in the bacterial clamp loader γ_3_δδ′ complex^43,44^. Thus far, the high-resolution structure of DNA-bound RFC–PCNA has not been achieved^45^, and the structure of a DNA-bound clamp–clamp loader complex is available only for the T4 bacterial phage system in which both the clamp loader and the clamp assume a spiral shape matching the helical symmetry of DNA^44^.

There are a few reported low-resolution EM observations of the human RAD17-RFC–9-1-1 and yeast Rad24-RFC–9-1-1 complexes, revealing the ring-like shapes of the 9-1-1 clamp and the loaders^46–49^. Owing to the lack of a high-resolution structure, it has been unclear whether the 9-1-1 clamp is indeed loaded onto the 5′-recessed DNA junction and, if so, how Rad24-RFC could load the 9-1-1 clamp ring in the opposite direction of the RFC loading of PCNA? Here we describe the first two structures of the Rad24-RFC–9-1-1 clamp bound to DNA and nucleotides, in closed and open conformations, at a resolution of 3.2 Å. Our structures reveal a surprising DNA binding mode, explaining the unique 9-1-1 clamp loading mechanism that is drastically different from that of PCNA loading by RFC.

## Results

### Assembly and cryo-electron microscopy of the Rad24-RFC–9-1-1–DNA complex

**Extended Data Fig. 1:**
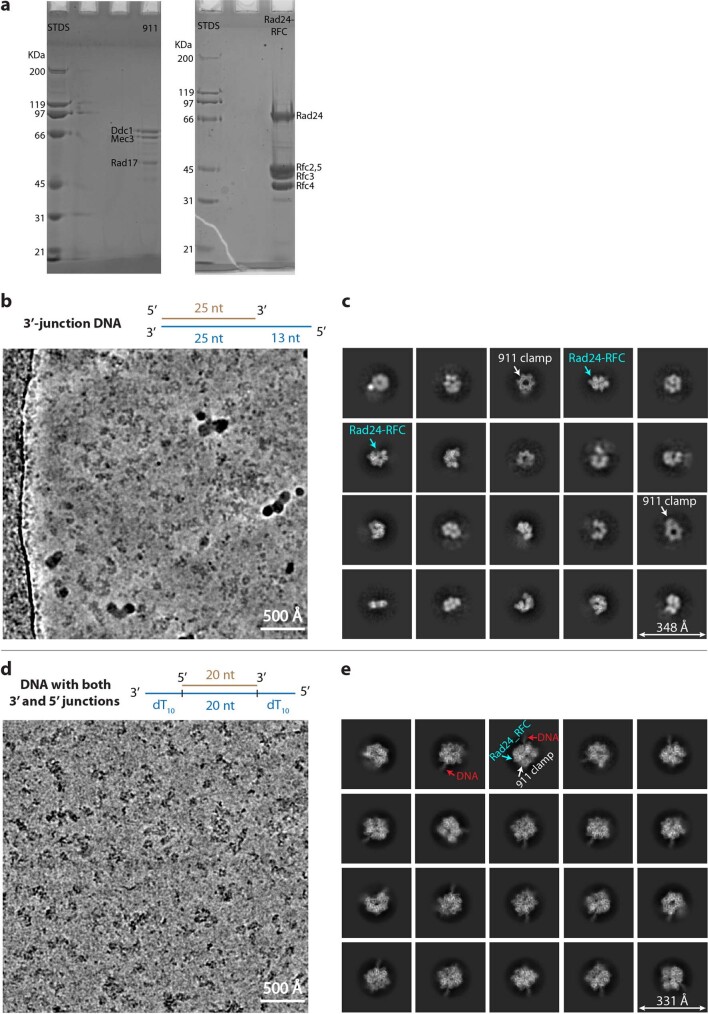
Assembly of the Rad24-RFC–9-1-1–DNA ternary complexes. **a)** SDS-PAGE gels of purified Rad24-RFC and the 9-1-1 clamp. Similar gels were run >3 times. **b**) A raw micrograph of the mixture of Rad24-RFC with 9-1-1 and a 3′-recessed DNA substrate after incubation in an ice-water bath for 3 hr. 12,000 similar micrographs were recorded. **c**) 2D class averages. No ternary complex of Rad24-RFC–9-1-1–DNA was observed. Only the separate 9-1-1 ring and the Rad24-RFC particles were observed. **d**) A typical raw micrograph of the mixture of Rad24-RFC with 9-1-1 and a double-tailed DNA substrate containing both the 5′- and 3′- recessed DNA junctions. 14521 similar micrographs were recorded. **e**) Selected 2D class averages with different views, revealing the presence of the targeted Rad24-RFC–9-1-1 clamp–DNA ternary complexes. Scale bars are shown in the lower right corner in each panel. Source data

**Extended Data Fig. 2:**
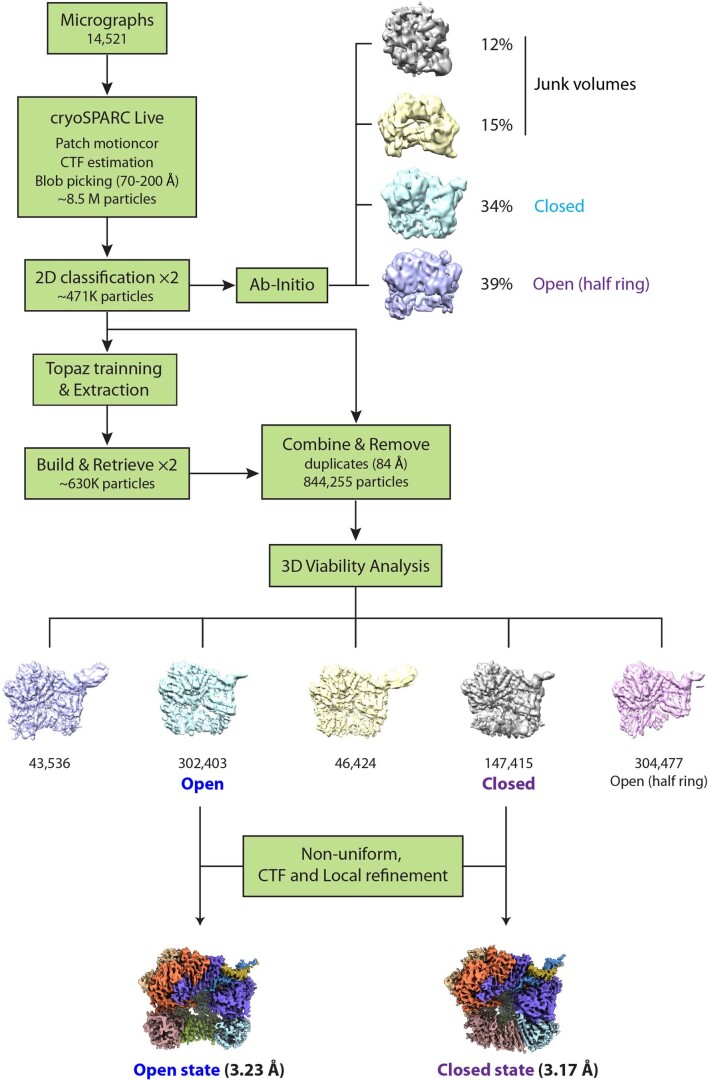
Workflow of image processing and 3D reconstruction. CryoSPARC Live is used to monitor data collection and real-time data processing. We used the particle picking program Topaz and ‘Build and Retrieve’ to obtain particles in more views. 3D variability analysis was applied to yield the 3.2-Å resolution 3D maps in the closed and open states of the Rad24-RFC–9-1-1 clamp–DNA complex.

**Extended Data Fig. 3:**
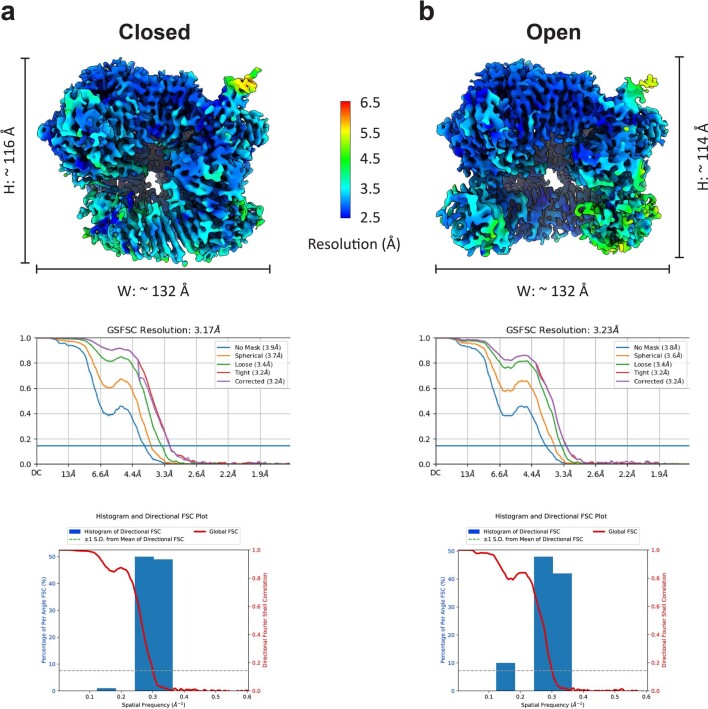
Resolution estimations of the closed (a) and open (b) states of the Rad24-RFC–9-1-1–DNA complex. Upper panels, the two 3D maps colored by local resolution. The dimensions of the structures are labeled. Middle panels, the 0.143 criterion of the gold standard Fourier shell correlation (GSFSC) was used to estimate the average resolutions. Bottom panels, the directional anisotropy of the two maps as quantified by the 3D-FSC server (https://3dfsc.salk.edu/). The sphericity of closed and open state is 0.949 and 0.923, respectively, demonstrating good anisotropic property of both maps.

**Extended Data Fig. 4:**
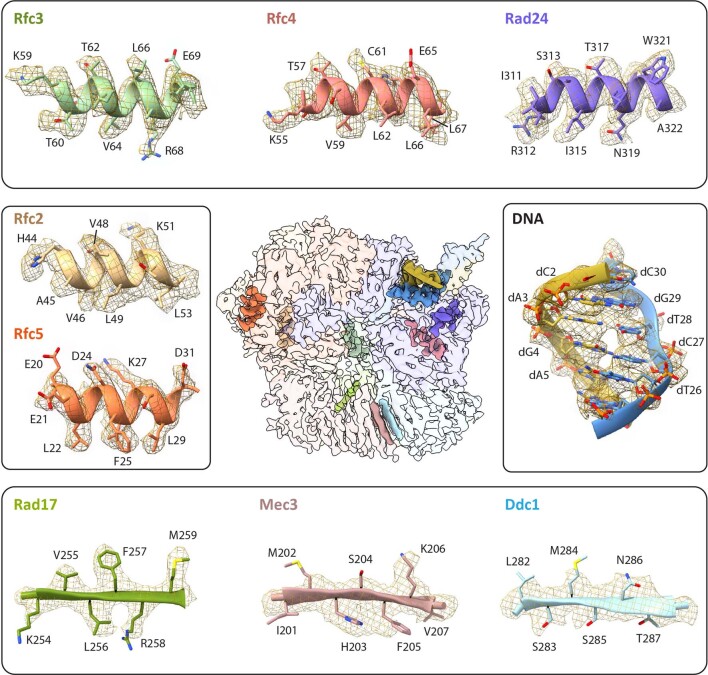
Fitting of the atomic model with the EM density in selected regions of the Rad24-RFC–9-1-1 clamp–DNA complex 3D maps. The central panel shows the segmented 3D map in 90% transparent surface view, except for the nine selected regions shown in solid surface. The nine selected regions – one from each component of the ternary complex – are shown in peripheral panels, with the atomic model in cartoon and residues in sticks, and with the EM density shown in wire meshes. For a fuller representation, the selected upper regions (Rad24-RFC–DNA) used the EM map in the open state, and the selected lower 9-1-1 clamp regions are from the EM map in the closed state.

We expressed and purified the *S.c.* Rad24-RFC and *S.c.* 9-1-1 clamp by recombinant means in *Escherichia coli* (Extended Data Fig. 1a). We first examined whether a 3′-recessed DNA substrate could induce the assembly of the Rad24-RFC–9-1-1–DNA ternary complex, but observed no complex (Extended Data Fig. 1b,c). We next designed a two-tailed DNA substrate in which both a 3′-junction and a 5′-junction are present (Fig. 1b). This DNA substrate enabled us to determine, in an unbiased manner, which recessed end Rad24-RFC prefers to bind. We mixed purified proteins with the DNA and 0.5 mM ATPγS, a slowly hydrolyzable ATP analog. Cryo-EM analysis indicated assembly of the Rad24-RFC–9-1-1–DNA ternary complex (Extended Data Fig. 1d,e). Subsequent two-dimensional (2D) and 3D classification, 3D reconstruction and 3D variability analysis led to two conformations of the ternary complex at an average resolution of 3.2 Å (Table 1 and Extended Data Figs. 2–4). In one conformation the 9-1-1 ring is closed, but in the other the ring is wide open with a 27-Å gap (Fig. 1c and Supplementary Video 1). The Rad24 structure was built de novo while modeling of Rfc2–5 referenced the shared subunits in the RFC–PCNA crystal structure^37^. The EM density of the 9-1-1 ring had a slightly lower resolution of ~3.8 Å, with Rad17 having the best density (Extended Data Fig. 3). The yeast 9-1-1 subunits contain long and disordered insertion loops that are larger than yeast PCNA (276 residues) or human 9-1-1^18^, but they all have a conserved core structure, facilitating modeling of the yeast 9-1-1 core structure^37,38^. The EM density of the DNA substrate was of sufficiently high resolution to distinguish purine from pyrimidine, enabling atomic modeling and unambiguous sequence assignment (Extended Data Fig. 4).

**Table 1:** Cryo-EM data collection, refinement and validation statistics

	Closed state (EMDB-25121, PDB 7SGZ)	Open state (EMDB-25122, PDB 7SH2)
**Data collection and processing**		
Magnification	105,000	105,000
Voltage (kV)	300	300
Electron exposure (e^−^/Å^2^)	65	65
Defocus range (μm)	1.1–1.9	1.1–1.9
Pixel size (Å)	0.828	0.828
Symmetry imposed	C1	C1
Initial particle images (no.)	844,255	844,255
Final particle images (no.)	147,415	302,403
Map resolution (Å)	3.17	3.23
FSC threshold	0.143	0.143
Map resolution range (Å)	2.0–10.0	2.0–13.0
**Refinement**		
Initial model used (PDB code)	1SXJ, 3G65	Closed state of this study
Model resolution (Å)	3.16	3.20
FSC threshold	0.143	0.143
Model resolution range (Å)	2.0–10.0	2.0–13.0
Map sharpening *B* factor (Å^2^)	−71.6	−62.8
Model composition		
Nonhydrogen atoms	21,056	20,934
Protein/DNA residues	2,558/23	2,545/23
Ligands	10	9
*B* factors (Å^2^)		
Protein/DNA	77.50/97.75	67.41/122.77
Ligand	48.03	45.52
R.m.s. deviations		
Bond lengths (Å)	0.002	0.002
Bond angles (°)	0.537	0.560
Validation		
MolProbity score	1.77	1.73
Clashscore	7.24	7.78
Poor rotamers (%)	0.04	0.04
Ramachandran plot		
Favored (%)	94.55	95.59
Allowed (%)	5.45	4.41
Disallowed (%)	0	0

In the atomic model of the Rad24-RFC–9-1-1–DNA complex, the five clamp loader subunits are arranged counterclockwise—Rad24-Rfc4-Rfc3-Rfc2-Rfc5 when viewed from the N-terminal AAA+ domains (Fig. 1d)—and, importantly, only the 5′-recessed end-containing half of the DNA was visible and stably bound to Rad24-RFC (that is, the 3′-recessed-end-containing half of the DNA was not visible). The resolved DNA includes 10 bp of the 5′-recessed DNA junction and three ssDNA nucleotides of the 3′-overhang (Fig. 1b,d). The DNA was mainly held by the AAA+ domain of Rad24, which differs completely from DNA binding in the inner chamber of all five subunits in the clamp loader–DNA complex that loads polymerase clamps^50^ (Fig. 1d,e). Because our DNA substrate contains both 3′- and 5′-recessed ends, the fact that Rad24-RFC bound only to the 5′-recessed half of the substrate confirms the reported preference of the Rad24-RFC (RAD17-RFC in human) to load 9-1-1 onto a 5′-recessed DNA end^11,13,21^, and it appears to do so even in the absence of RPA under the conditions used here.

### Unique Rad24 features recognizing the 5′-recessed DNA junction

**Fig. 2:**
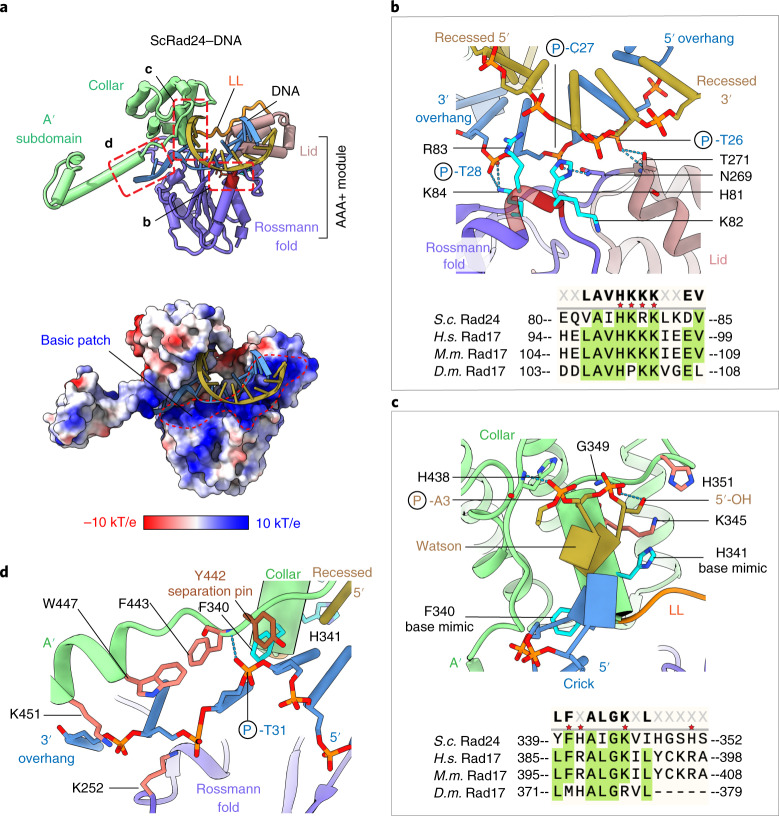
Rad24 interaction with the 5′-recessed DNA junction. **a**, Top: structure of Rad24 bound to the DNA in cartoon view. The 5′-strand is orange and the 3′-strand is cyan. Residues 81-HKRK-84 of the AAA+ domain are in dark red. Areas marked by three dashed red rectangles are shown as close-up views in **b**–**d**, respectively. Bottom: electrostatic surface view showing a contiguous DNA-binding basic patch on the top of the AAA+ domain. LL: long linker between AAA+ and the collar domains that enables the formation of a large groove to accommodate the DNA. **b**, Top: interaction between the AAA+ domain and DNA. The four tandem basic residues are shown as cyan sticks. His-81 and Arg-83 insert into the DNA minor groove, and Lys-84 forms two hydrogen bonds with the DNA T28 phosphate of the 3′-strand. Asn-269 of the AAA+ domain and Thr-271 of the AAA+ domain form three hydrogen bonds with the DNA phosphates of C-27 and T-26. Bottom: sequence alignment of the Rad24 four tandem basic residues. *S.c.*, *Saccharomyces cerevisiae*; *H.s.*, *Homo sapiens*; *M.m.*, *Mus musculus*; *D.m.*, *Drosophila melanogaster*. **c**, Top: DNA lies on the Rad24 AAA+ domain. Phe-340 and His-341 of the collar domain stabilize the 5′-recessed DNA junction and mimic a nucleotide base to form a hydrophobic stack with the last base pair at the 5′-junction; they each rotate 32° with respect to the interacting base, resembling the 36° rotation of a normal base. His-341, Lys-345 and His-351 surround the 5′-OH of the 5′-strand and prevent a 5′-overhang from binding there. The 5′-OH and the A-3 phosphate form hydrogen bonds with Gly-349 and His-438, respectively. Bottom: sequence alignment of Rad24 base-mimicking residues Phe-340 and His-341, and the 5′-OH blocking Lys-345, His-351. **d**, Residues guiding 3′-overhang ssDNA into the Rad24-RFC chamber. Tyr-442 is positioned at the 5′-recessed DNA junction resembling a separation pin.

**Extended Data Fig. 5:**
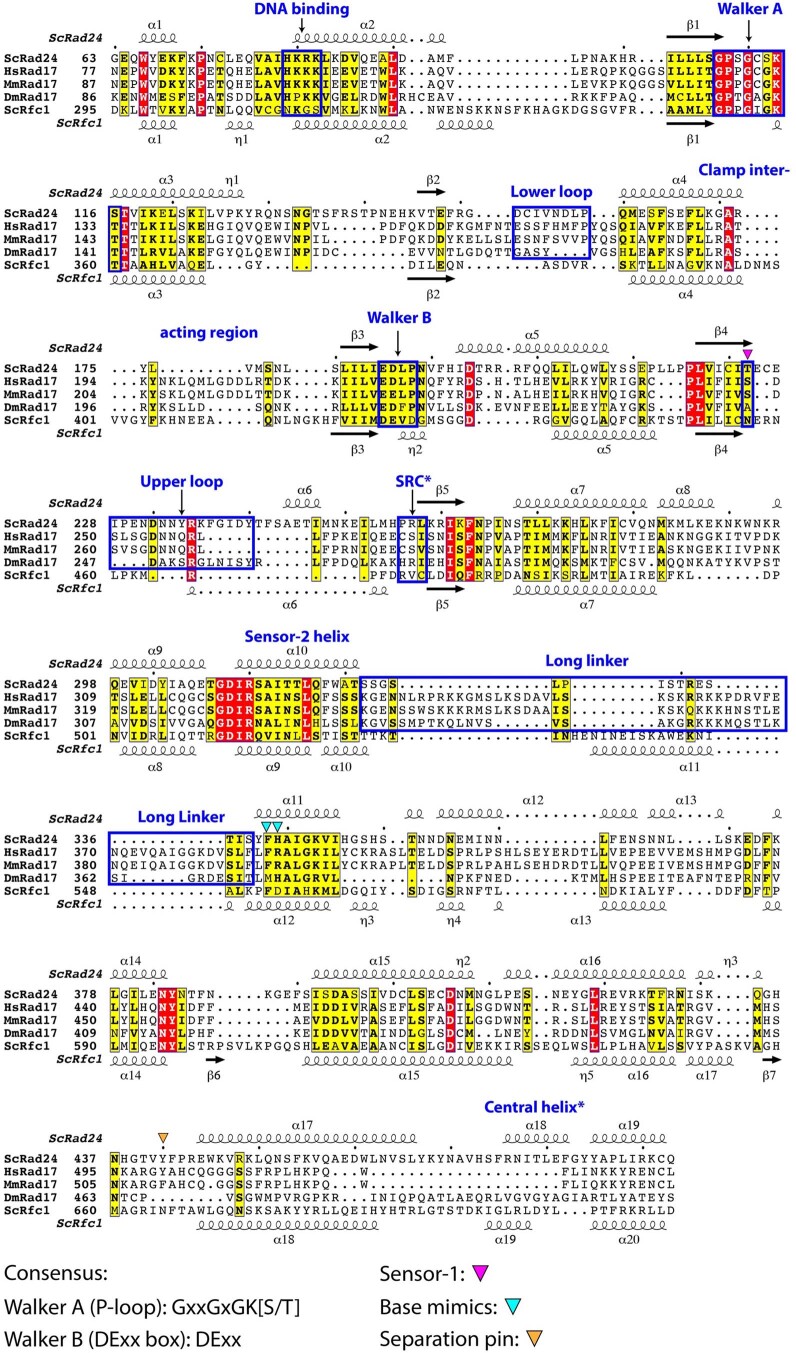
Sequence alignment of yeast Rad24, metazoan RAD17 and yeast Rfc1. Helices and β-strands are shown as coils and arrows, respectively. The assignments are produced by ESPript 3.0 (https://espript.ibcp.fr/ESPript/ESPript/) based on the structures S.c. Rad24 (this study) and S.c. Rfc1 (PDB code 1SXJ). The flashy color scheme was applied. Key features are marked above the sequences. Note that the linker region between collar and AAA+ module of Rfc1 contains only one residue (L549) and is not included in the box. The SRC (serine-arginine-cysteine) motif – conserved in Rfc2–5 – is absent in Rad24/RAD17 and Rfc1/RFC1; its location corresponding to the SRC motif in Rfc2–5 is boxed and labeled as SRC*.

**Extended Data Fig. 6:**
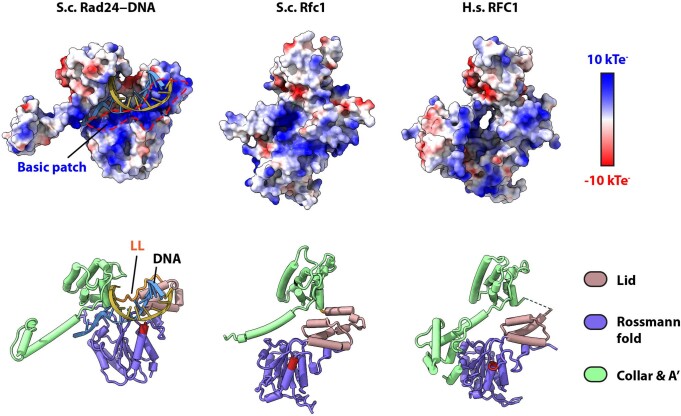
Structural comparison of Rad24 and Rfc1/RFC1. Upper panel, the electrostatic potential charge surface of the S.c. Rad24 (this study), S.c. Rfc1 (PDB code 1SXJ), and human RFC1 (PDB code 6VVO). In Rad24, a contiguous basic patch on top of the AAA+ domain enables DNA binding. Rfc1/RFC1 lack such contiguous basic path. Lower panel, the corresponding atomic models shown in cartoons. These structures are aligned based on the full loader complexes to achieve the best superposition. The four tandem basic residues in Rad24 and the corresponding region in Rfc1/RFC1 are highlighted in firebrick. Note that the lid and Rossmann fold domains move away from the collar domain plus the associated A’ domain by a 35-Å shift and a 20º rotation, creating a large groove for dsDNA binding.

The open and closed complex structures are similar in the loader region, with the main difference being the 9-1-1 ring structure. Here we choose the closed state to illustrate the DNA binding with Rad24 in the loader (Supplementary Video 2). The most striking feature is that the DNA is bound only by the Rad24 subunit in an extended groove on its AAA+ module and extending to the collar domain (Fig. 2a). Several features of Rad24 have apparently enabled the unique DNA binding mode in Rad24-RFC. The first is the 15-residue ‘long linker’ (sometimes referred to here as ‘LL’) from residue 324 to residue 338 that connects the Rad24 AAA+ module to the collar domain (Figs. 1a and 2a). The long linker has enabled regions of the AAA+ module to move 35 Å and rotate 20° away from the Rad24 collar domain, forming the DNA-binding cleft (Supplementary Video 3). This linker is one residue long in Rfc1, the Rad24 equivalent of the RFC complex^37^, and is 21 residues long (residues 827 to 847) but disordered in hRFC1^38^ (Extended Data Fig. 5). In both *S.c.* Rfc1 and hRFC1, the collar domain is next to the AAA+ domains, so no DNA binding groove could exist without major conformational changes (Extended Data Fig. 6).

The second important feature of Rad24 is the presence of a long and contiguous basic patch including 81-HKRK-84 on the top of the AAA+ domain, enabling stable DNA binding (Fig. 2a,b). Such a continuous basic patch is absent in *S.c.* Rfc1 and hRFC1 (Extended Data Fig. 6). The side chains of His-81 and Arg-83 insert into the DNA minor groove, and Lys-84 forms two hydrogen bonds with the T-28 phosphate of the 3′-strand. Lys-82 points away from the DNA but contributes to the overall positively charged environment of the DNA patch. In addition, Asn-269 of the AAA+ domain and Thr-271 of the AAA+ domain contact the DNA, forming hydrogen bonds with the phosphate groups of C-27 and T-26 of the 3′-strand. The four residues within the basic patch are largely conserved among the 9-1-1 loading eukaryotic Rad24/RAD17-RFCs, but are not present in the PCNA loading Rfc1-RFC (Fig. 2b and Extended Data Fig. 5).

At the 5′-junction, the aromatic Phe-340 and His-341 in the Rad24 α-helix 11 form hydrophobic stacking interactions with DNA residues C-30 and G-1 (Extended Data Fig. 5), respectively, thereby stabilizing the last base pair, G-1:C-30 (Fig. 2c). These two aromatic side chains are rotated ~32° relative to the last two bases (G-1 and C-30), mimicking the normal 36° rotation of base pairs in dsDNA. In vitro synthesized DNA in previously published studies of 9-1-1 clamp loading used 5′-OH DNA, so we also used 5′-OH DNAs^11,13^. Importantly, we observed that the G-1 5′-OH of the DNA in our structure is surrounded by three positively charged residues, His-341, Lys-345 and His-351. These residues are well positioned to neutralize the phosphate at the 5′-P-DNA junction and thus possibly even prevent a 5′-ssDNA overhang from binding at the site. The 5′-junction is further stabilized by a hydrogen bond between Gly-349 of the collar domain and 5′-OH of DNA and by another hydrogen bond between His-438 of the A′ subdomain of Rad24 and the A-3 phosphate of the DNA (Fig. 2c). These structural features probably account for Rad24’s specificity for the 5′-recessed DNA junction^11,13^. The DNA-interacting residues are conserved among yeast Rad24 and metazoan RAD17 (Fig. 2b,c).

Rad24 also stabilizes the first three nucleotides (T-31, T-32 and T-33) of the 3′-overhang (Fig. 2d). Specifically, Tyr-442, structurally equivalent to the ‘separation pin’ Tyr-316 in the *E. coli* clamp loader^50^, is positioned right at the 5′-junction of the ssDNA and dsDNA. Phe-443, Trp-447 and Lys-451 from the Rad24 A′ subdomain then stabilize the exposed bases of the three 3′-overhang nucleotides. Finally, Lys-252 forms an electrostatic interaction with the DNA T-32 phosphate, and the main chain nitrogen of Phe-443 forms a hydrogen bond with the DNA T-31 phosphate of the 3′-strand, guiding the 3′ ssDNA overhang towards the interior chamber of the Rad24-RFC loader.

### Rad24-RFC is poised to hydrolyze ATP

**Fig. 3:**
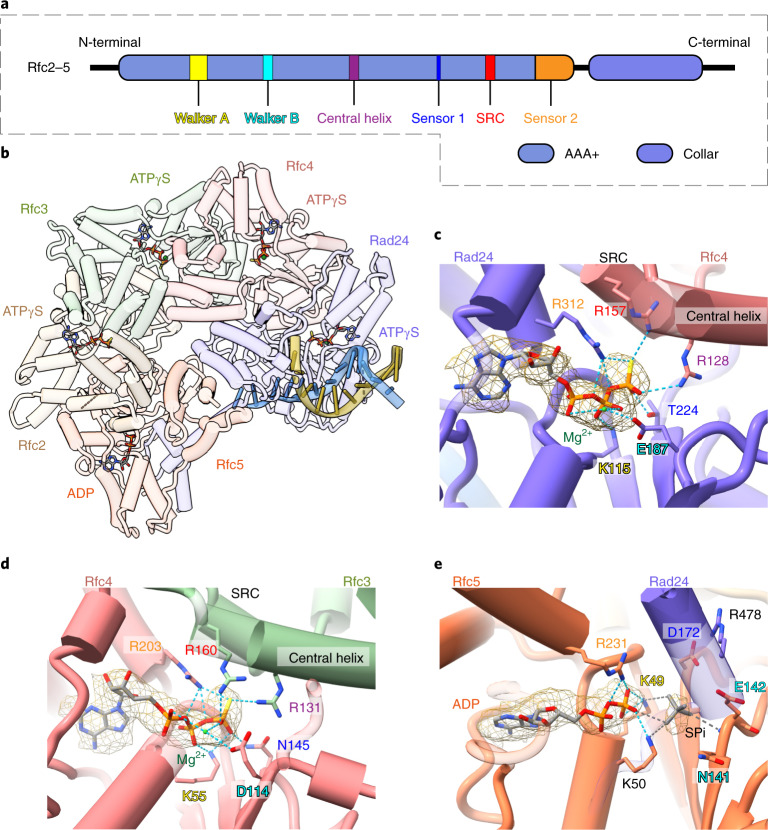
Four ATPγS and one ADP are bound to Rad24-RFC. **a**, Key ATPase motifs in Rfc2–5. Rad24 lacks the SRC and central helix. **b**, Top view of Rad24-RFC–DNA in cartoon, omitting the 9-1-1 clamp for clarity. The bound four ATPγSs and one ADP are shown as sticks. **c**–**e**, Enlarged views of the nucleotide binding pockets in Rad24 (**c**), Rfc4 (**d**) and Rfc5 (**e**). ATPγS binding in Rfc2 and Rfc3 is similar to that in Rfc4 and Rfc3 and is not shown. The residues of the AAA+ module involved in nucleotide binding are the conserved SRC (serine-arginine-cysteine) motif, sensor-1, Walker A (P-loop), Walker B (DExx box), Sensor-2 and the central helix; these are labeled and colored red, orange, yellow, cyan, blue and purple, respectively. The Mg^2+^ ion is in green. The Rfc5 nucleotide pocket is occupied by ADP. Because Rad24 lacks the SRC motif, there is only one arginine (Arg-478) at the Rfc5:Rad24 interface, which points away from the nucleotide due to the absence of the γ-phosphate. The isolated strong density (displayed at 6*σ*) next to ADP is modeled as a thiophosphate (SP_i_) and is shown in gray sticks. The phosphate is coordinated by both Lys-49 and Lys-50 of the Walker A motif as well as the backbone nitrogen of Glu-142 in the Walker B motif. For clarity, only the hydrogen bonds with the nucleotide phosphates are shown.

**Extended Data Fig. 7:**
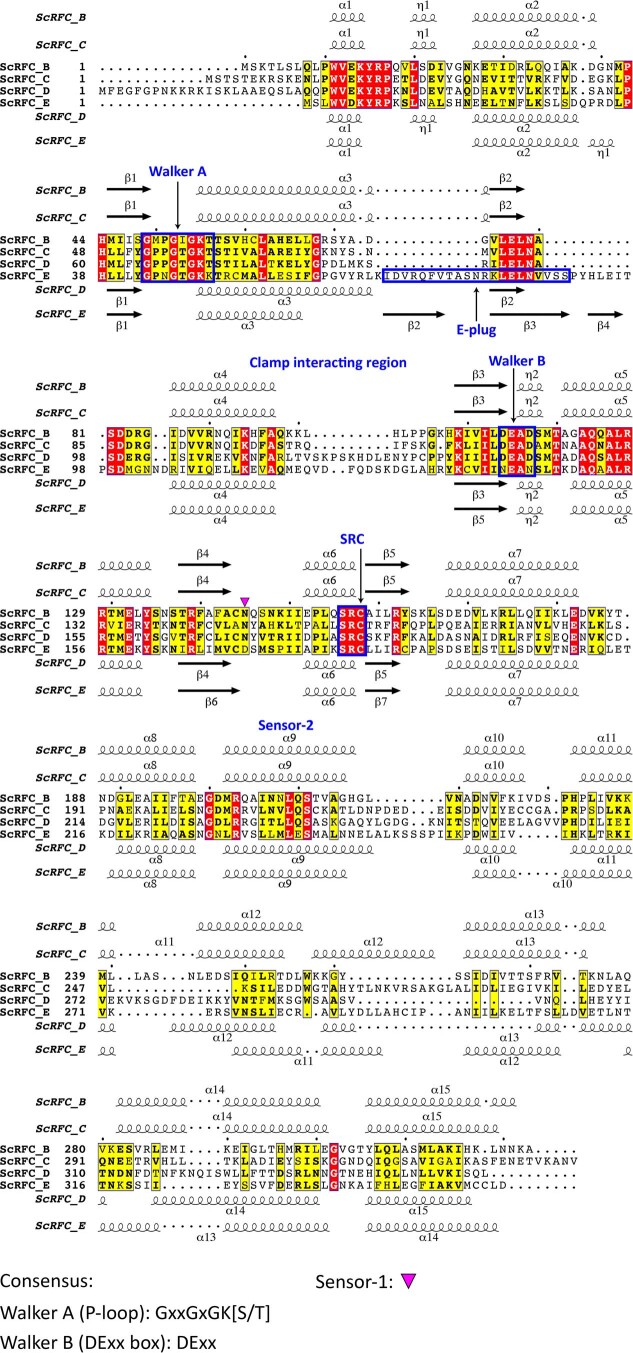
Structure-based sequence alignment of S.c. Rfc2–5. Structural elements of Rfc3 and 4 are shown above and that of Rfc2 and 5 are shown below the sequences. Assignment of the secondary structural elements also considered both the Rad24-RFC structure (current study) and the S.c. RFC structure (PDB code 1SXJ).

Each of the five nucleotide-binding sites of Rad24-RFC is occupied by a nucleotide. In both closed and open 9-1-1 clamp states, the first four interfaces (Rad24:Rfc4, Rfc4:Rfc3, Rfc3:Rfc2 and Rfc2:Rfc5) are occupied by ATPγS and Mg^2+^, and an adenosine diphosphate (ADP) occupies the last interface between Rfc5 and the A′ subdomain of Rad24 (Fig. 3a,b). This observation suggests that the 9-1-1 gate opening does not require ATP hydrolysis by Rad24-RFC. The ATP sites of Rad24 and Rfc2–4 have been shown to be required for efficient 9-1-1 loading^20^. It is remarkable that the nucleotide binding pattern resembles those in the RFC–PCNA structures, in which the first four interfaces are bound to ATPγS and the last interface to ADP^37,38^, and this is also the case in the T4 clamp–clamp loader–DNA structure^44^. Sequence alignment identified conserved AAA+ ATPase motifs such as the Walker A (P-loop; consensus: GxxGxGK[T/S], where x is any residue), Walker B (DExx box), Sensor 1 and Sensor 2 motifs^34,41^ (Fig. 3a and Extended Data Figs. 5 and 7). Starting from Rad24 and counterclockwise, in the first four nucleotide binding pockets, the Walker A motif (P-loop, see sequence alignment) wraps the triphosphate, and the P-loop lysine forms a hydrogen bond with the phosphate group; the first acidic D/E of the Walker B motif (DExx box) coordinates a Mg^2+^ ion and hydrogen-bonds with ATPγS, and the Sensor-1 residue T/N and the basic arginine residues from the Sensor 2 helix also hydrogen-bond with the phosphate group of ATPγS (Fig. 3c,d). The adjacent subunit also contributes to nucleotide binding. Unexpectedly, the two arginine residues from the central α5-helix and the SRC-containing α6-helix each form hydrogen bonds with the γ-phosphate in the Rad24-RFC–9-1-1–DNA structures, indicating that the first four nucleotide binding sites are poised for ATP hydrolysis, even though the Rad24-RFC is nearly planar (Fig. 3b–d). This is different from the spiral RFC–PCNA structure, in which only the ATP analog in the first interface between RFC1 and RFC2 is tightly bound. In the following three ATP sites of RFC–PCNA, the arginine finger residues of the SRC motifs are 8–17 Å away from the γ-phosphate, indicating an inactive ATPase state^38^. This difference between the nearly planar Rad24-RFC and spiral RFC structures is probably due to the presence of DNA in the Rad24-RFC structure, as DNA binding and the subsequent clamp loading probably induce conformation changes that could switch the loader from the inactive to active state, eventually triggering ATP hydrolysis and release of the loaded DNA clamp from the clamp loader^37,38,44^

The presence of ADP at the interface of Rfc5:Rad24 is remarkable, as this resembles those observed at the equivalent site in all three structurally determined clamp–clamp loader systems, despite the substantial differences between Rad24 and Rfc1^37,38,44^ (Fig. 3e). Rfc5 harbors ATPase motifs. However, at the opposing side of the interface, the Rad24 C-terminal A′ subdomain has only one helix (α-18) that harbors an arginine residue (Arg-478), as compared to two helices—the SRC-containing helix and the central helix—at all other ATP binding interfaces. The Walker A motif of Rfc5 (^43^GPNGTGK*K*T^51^) has an extra lysine (Lys-50)^38,51,52^ (Extended Data Fig. 7). A previous study found that mutation of the first lysine (Lys-49) alters neither the ATPase activity nor DNA replication activity of the RFC complex^51^, leading to the suggestion that this site is inactive in ATP hydrolysis and that the observed ADP may be co-purified with the loader or as a contaminant typically found in ATPγS preparations^20,38^. The adjacent Rfc5 Lys-50 also appears to be involved in ADP binding in our structure, suggesting a possible involvement in hydrolysis. We noted a bulky density near the ADP β-phosphate in our EM map when displayed at a high threshold of 6*σ* (Fig. 3d). This density is adequate to accommodate a phosphate and we cannot rule out that ATPγS might be hydrolyzed at this site.

### Interactions between Rad24-RFC and the 9-1-1 clamp

**Fig. 4:**
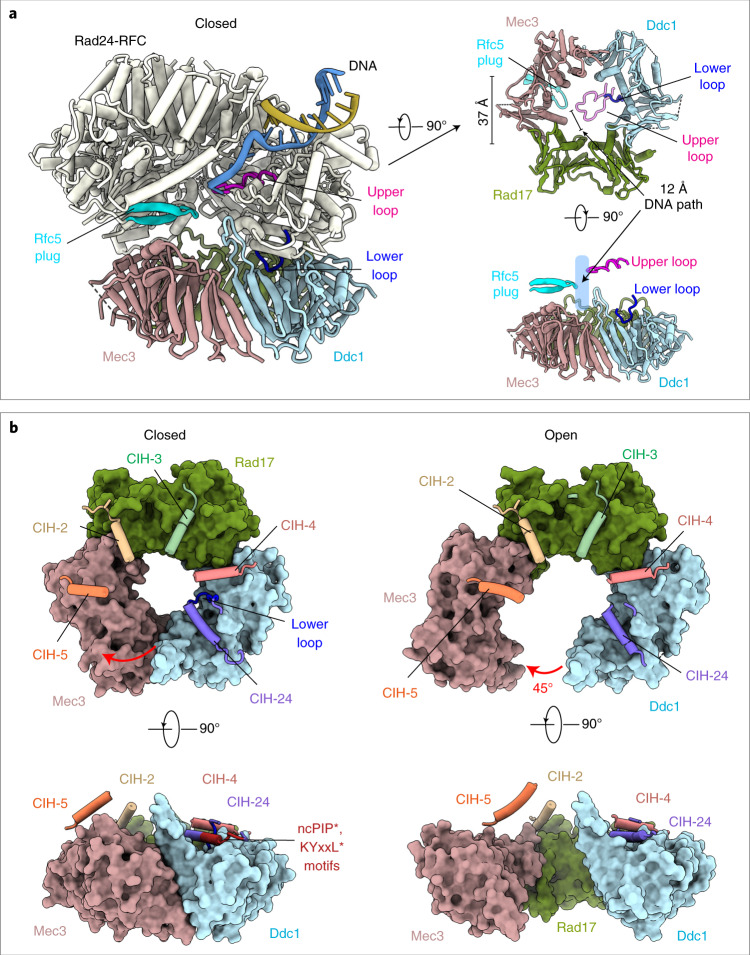
Interaction between Rad24-RFC and the 9-1-1 clamp in the closed and open states. **a**, Tilted, bottom and side views of the closed 9-1-1 clamp, highlighting the positions of three important loops. The upper insertion loop of Rad24 and the Rfc5 plug narrow the interior chamber of Rad24-RFC to 12 Å, suitable for ssDNA but excluding dsDNA binding. The Rad24 lower insertion loop binds Ddc1 of the 9-1-1 clamp to control the ring opening/closure. The inner diameter of 9-1-1 is 37 Å. **b**, Comparison of the closed and open 9-1-1 clamp in top and side surface views. Superimposed on the clamps (in surface view) are the CIH and connecting loop of each loader subunit, shown in cartoon view. The non-canonical PIP (ncPIP) motif and the KYxxL-like motif at the end of the Rad24 CIH are labeled and highlighted in dark red. The Rad24 lower loop bound to Ddc1 is ordered in the closed state but becomes disordered and loses contact with Ddc1 in the open state, leading to a 45° in-plane rotation of Mec3 to form the DNA entry gate.

Rad24-RFC features three insertion loops that are important to binding DNA and the 9-1-1 clamp (Fig. 4a). The first loop is a long insertion in the AAA+ domain of Rad24, termed the upper loop. The second loop is an insertion in Rfc5, which we call the Rfc5 plug, which is much longer than those of Rfc2–4 (Extended Data Fig. 7). These two loops project into the interior chamber of Rad24-RFC and constrain the DNA path to 12 Å, which is narrower than the 20-Å dsDNA width. We thus propose that these two loops prevent dsDNA binding to the inner chamber of Rad24-RFC and thus facilitate the inverted DNA binding mode in Rad24-RFC. The third loop is hook-like, projecting from the Rad24 AAA+ domain, and interacts with Ddc1 of the 9-1-1 clamp in the closed state. This loop becomes disordered in the clamp open state (Fig. 4a,b). This clamp binding feature is found only in the Rad24 family of proteins and is absent in the RFC complexes (Extended Data Fig. 5). We propose that this loop stabilizes the open clamp structure, but, due to the resolution in this region of Ddc1, we cannot make a definitive assignment.

Interactions between Rad24-RFC and 9-1-1 are primarily mediated by the clamp interacting α4-helices (CIHs) and the loops that follow them, and these interactions are similar to those between RFC and PCNA^37,38^ (Fig. 4a,b and Extended Data Figs. 5 and 7). However, only three RFC subunits were bound to PCNA, because the PCNA ring and the RFC spiral are at an angle. By contrast, all five subunits of Rad24-RFC had some contacts with the 9-1-1 clamp in both closed and open states due to the planar structure of both complexes, and thus the 9-1-1 and the Rad24-RFC rings are nearly parallel to each other, enabling interactions between the ring and all five clamp loader subunits. In the closed conformation, Rad24 sits above Ddc1, having the largest interface of 830 Å^2^. However, this interface is weakened and reduced by one-third in the open state. The interface between Rfc2 and Ddc1 is 400 Å^2^ in the closed state and is reduced slightly to 350 Å^2^ in the open state. The interfaces between Rad17 and Rfc3 and between Rad17 and Rfc4 are stable, with a total area of 940 Å^2^ in both closed and open states. Compared to the buried surfaces of 2,000, 700 and 1,200 Å^2^ between PCNA and RFC1, RFC2 and RFC3, respectively^38^, the binding between Rad24-RFC and 9-1-1 would appear to be substantially weaker.

Overall, Rad24 contributes the most binding affinity for the 9-1-1 clamp. Rad24 lacks the canonical PCNA-interacting peptide (PIP) motif^53^. However, sequence alignment suggests a non-canonical PIP motif ^169^FLKGARYL^176^ in the AAA+ domain, and this motif interacts with the 9-1-1 clamp (Fig. 4b and Extended Data Fig. 5). Interestingly, the last three residues of the non-canonical Rad24 PIP motif plus the following two residues (^174^RYLVM^178^) are equivalent to the KYxxL motif in hRAD17 that was previously shown to be essential for 9-1-1 binding^54^; indeed, this five-residue motif also contributes to the yeast 9-1-1 clamp binding in our structure.

### Both the open and closed 9-1-1 clamps are planar

The Rad24-RFC loader is configured into a two-tiered spiral, with the upper tier formed by the helical ‘collar’ domain and the lower tier by the AAA+ ATPase module, composed of two domains, that engages the clamp ring (Figs. 1d and 4a). The Rad24-RFC complex conformation is essentially unchanged in the 9-1-1–DNA open and closed states (Extended Data Fig. 3a,b). The structures are only differentiated by a root mean square deviation (r.m.s.d.) of 1.0 Å when the two gap-lining 9-1-1 subunits Ddc1 and Rad17 are excluded from comparison (Fig. 4b). We found that Mec3 of the closed form undergoes a 45° in-plane rotation away from Ddc1 to generate the 27-Å gap in the open form of the 9-1-1 clamp (Supplementary Video 1). This is the largest gap observed so far in any DNA clamp-loader complex structure; the previously observed open clamp rings only have a gap of 10 Å or less that is too narrow for dsDNA to pass through^37,38,44,45,49^. Importantly, the gate opening coincides with the above-mentioned disordering and perhaps unbinding of the Rad24 lower insertion loop (Fig. 4b). Therefore, we suggest that this Rad24 loop controls the 9-1-1 ring opening.

**Extended Data Fig. 8:**
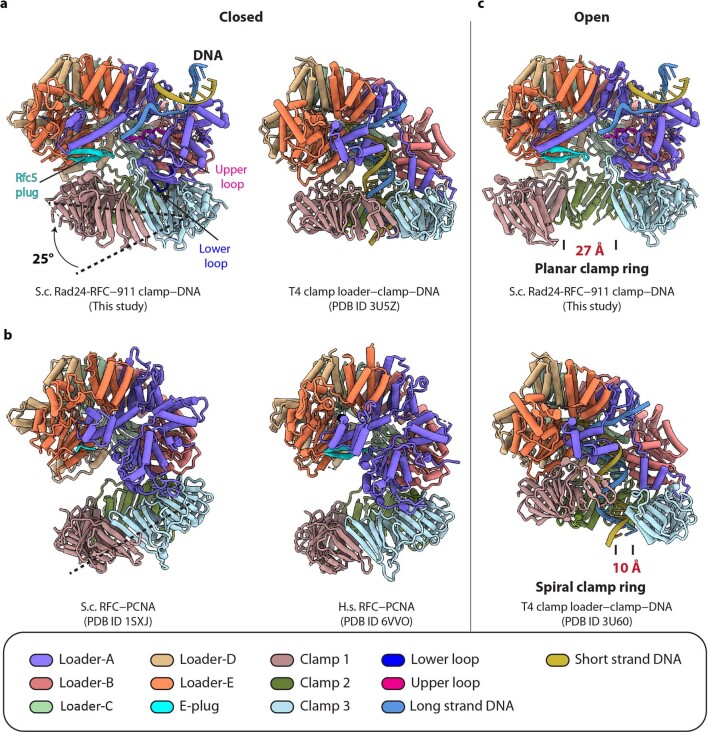
Comparison of the available structures of the DNA clamp–clamp loader–(DNA) complexes. **a**) Structure of the yeast Rad24-RFC bound to 9-1-1 and DNA compared with the T4 phage clamp loader–clamp bound to a DNA substrate. **b**) Comparison of the yeast RFC–PCNA (PDB code 1SXJ) with the human RFC–PCNA (PDB code 6VVO), all in a clamp ring closed configuration. The Rad24-RFC–9-1-1 clamp is more compact than the yeast and human RFC–PCNA structures, with the 9-1-1 ring tilting 25° toward the Rad24-RFC loader. **c**) Structure comparison of the Rad24-RFC–9-1-1 clamp–DNA with the T4 clamp–clamp loader–DNA (PDB code 3U60), both in the open clamp ring conformation. Note the 27-Å DNA gate in the planar 9-1-1 clamp with the 10-Å DNA gate in the T4 spiral clamp. To facilitate comparison, the loader subunits are labeled A through E according to their physical location in the complex.

The pre-existing RFC–PCNA structures were determined in the absence of a DNA substrate in which the PCNA is a closed ring, and the RFC loader is spiral^37,38^. Compared with the RFC–PCNA structure, the 9-1-1 clamp tilts up by 25° to engage the loader in our DNA-bound Rad24-RFC–9-1-1 structure (Extended Data Fig. 8 and Supplementary Video 1). Furthermore, the Rad24-RFC loader is wider than the RFC loader due to a large expansion of the Rad24 structure to accommodate the DNA. However, the close approach of the 9-1-1 ring towards Rad24-RFC resembles the DNA-bound T4 phage clamp–clamp loader structure^44^. Strikingly, the 9-1-1 ring is planar in both the closed and open states of the Rad24-RFC–9-1-1 clamp–DNA complex. This contrasts with the spiral T4 clamp loader–clamp with DNA^44^. Although the physiological role of maintaining a planar 9-1-1 ring during the DNA loading process is currently unclear, we speculate that this unique feature may be related to the fact that 9-1-1 is loaded onto ssDNA, not dsDNA, and then slides to the 5′-recessed junction after it is loaded and separates from the Rad24-RFC complex.

## Discussion

### Model of clamp loader function

**Fig. 5:**
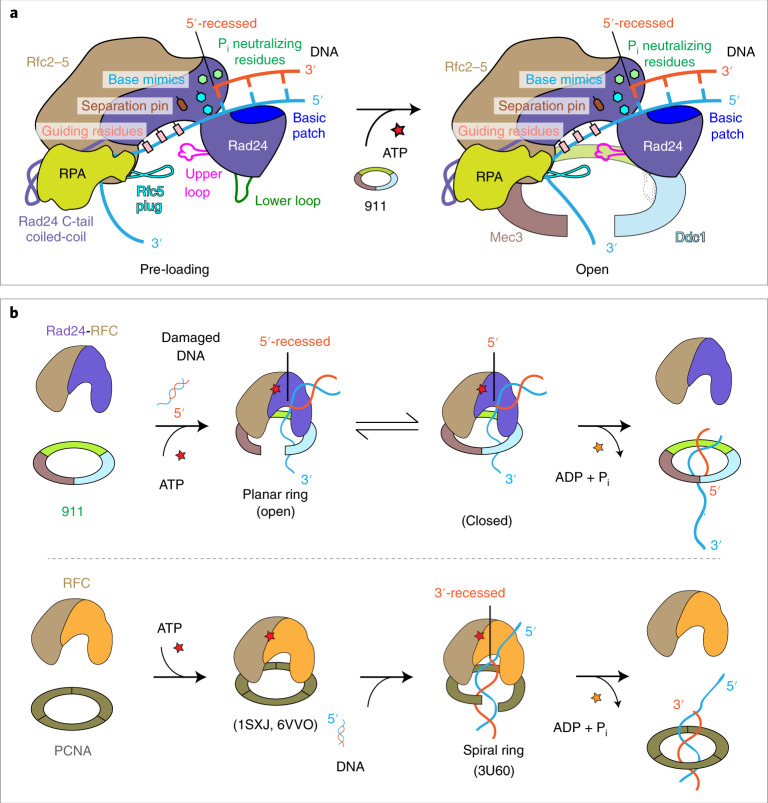
The mechanism of 9-1-1 loading onto the 5′-recessed DNA junction by Rad24-RFC. **a**, Rad24 confers the 5′-recessed DNA junction with specificity via several unique structural features: the long linker between the collar and AAA+ domains, enabling their separation and the formation of the dsDNA binding groove; the basic patch on top of the AAA+ domain that binds dsDNA; the 5′-phosphate neutralizing residues, the base mimics, the separation pin that facilitates binding to the 5′-junction; and the Rad24 upper loop and the Rfc5 plug, which limit the chamber size and prevent dsDNA from entering the loader chamber. RPA binds to the C-terminal coiled coil of Rad24, thereby holding the ssDNA of the 5′-recessed DNA junction away from the space below the Rad24-RFC where the 9-1-1 clamp will bind. This is a bipartite DNA binding mechanism that we propose facilitates 9-1-1 clamp recruitment and loading onto ssDNA at a 5′ DNA junction. The loaded 9-1-1 clamp could then diffuse from ssDNA onto the dsDNA. **b**, Upper panel: the loading steps of the 9-1-1 clamp by Rad24-RFC. Both open and closed states are observed in this study. Lower panel: the loading steps of PCNA by RFC based on previous structural studies. In addition to the opposite DNA binding mode, the inner chamber of RFC is a spiral that is molded by the binding of DNA, whereas Rad24-RFC does not adopt a spiral structure as the dsDNA region is solely bound by the Rad24 subunit and does not enter the inner chamber of Rad24-RFC. See main text for details. PDB codes for the structures are indicated (1SXJ, 6VVO and 3U60).

The ssDNA binding protein RPA is reported to be involved in Rad24-RFC function and its specificity for the 5′-junction^13^. This function is mediated by a protein-protein interaction between the 180-residue N-terminal domain of Rpa1 and the C-terminal coiled-coil domain (amino acids (aa) 473–652) of Rad24^13,55^. The Rad24 C-terminal coiled coil is disordered in our structure determined in the absence of RPA, but its approximate location can be predicted following the visualized A′ subdomain to which the coiled coil is appended. Based on our structure, we suggest that RPA–Rad24-RFC together provide a bipartite damaged DNA binding platform, with Rad24-RFC binding at the 5′-junction and the RPA binding to the 3′ ssDNA overhang before the 5′-recessed DNA junction (Fig. 5a, left). Such bipartite binding probably holds the 3′-overhang ssDNA away from the space below the AAA+ ring of Rad24-RFC, thereby clearing the way for 9-1-1 to engage Rad24-RFC on the 5′-recessed DNA junction. We speculate that RPA may further align the 3′-overhang ssDNA from the 5′-recessed DNA junction with the open gate of the 9-1-1 clamp, in a manner akin to Orc5-6 of the origin recognition complex (ORC) holding one end of the replication origin DNA away from the Mcm2–7 binding space and aligning the DNA with the Mcm2-Mcm5 DNA gate of the Cdt1-bound Mcm2–7^56–58^.

Although RPA appears to be the first responder to DNA damage, it acts together with Rad24-RFC to mount an effective response. We suggest that RPA holds the 3′-overhang ssDNA at a 5′-recessed DNA junction, and Rad24-RFC specifically recognizes the 5′-junction and binds the dsDNA region between the collar domain and AAA+ domain of Rad24. The 5′-junction recognition is probably mediated by a number of unique Rad24 residues, including the three residues that neutralize the 5′-phosphates (that is, His-341-, Lys-345 and His-351), the DNA separation pin at the junction (Tyr-442) and the two base-mimicking residues (that is, Phe-340 and His-341) that may stabilize the last base pair at the junction (Fig. 5a, left).

Next, Rad24-RFC recruits the 9-1-1 clamp to the DNA damage site via the five CIHs, one from each loader subunit, and then inserts a hook-like loop at the bottom surface of the Rad24 AAA+ domain into the 9-1-1 clamp and binds exclusively to the Ddc1 clamp subunit (Fig. 4b, left). This binding event on Ddc1 probably triggers the neighboring Mec3 clamp subunit to undergo an in-plane rotation away from Ddc1, generating a gap wide enough to admit either an ssDNA or a dsDNA. Loading of the 9-1-1 on the DNA will presumably stimulate ATP hydrolysis by Rad24-RFC, leading to ring closure of 9-1-1 and separation from Rad24-RFC. The ATP hydrolysis separation mechanism is supported by structural analysis of the T4 clamp loader system in which ATP hydrolysis results in a conformation that is incompatible with clamp binding (Extended Data Fig. 8)^44^. Departure of the clamp loader exposes the 9-1-1 ring for recruitment of downstream checkpoint proteins to the damage site for DNA repair, such as the ATR kinase (yeast Mec1-Ddc2)^21^.

The mechanism of PCNA loading on DNA by the eukaryotic RFC has been studied extensively^34,40^. Structures of RFC–PCNA in the absence of DNA indicate that RFC first engages PCNA, then RFC–PCNA engages the 3′-recessed DNA junction, leading to PCNA loading and encircling the dsDNA^37,38^ (Fig. 5b, bottom). There is one reported structure of the clamp–clamp loader complex bound to a DNA substrate of the T4 phage system^44^. In that structure, the 3′-recessed DNA junction has entered the inner chamber, but the 5′-overhang is extruded from the chamber (Extended Data Fig. 8a,c). That structure may represent the post-loading state. A larger gate opening may have been available to admit the dsDNA, but the gate is partially closed once the DNA has entered. Another possibility is that the clamp may initially be loaded onto ssDNA and then the 3′-recessed junction may slide into the clamp^37,44^.

### Why is the gap in the 9-1-1 ring so large?

Although we can only speculate on the function of the large gap in open 9-1-1, there are some reasonable possibilities. First, the large opening of the 9-1-1 clamp may assist loading in the presence of RPA bound to the 3′ ssDNA overhang. Second, future study of the entire loading mechanism might show an intermediate where dsDNA must fit through the gap in the 9-1-1 ring. Third, there may be no selection pressure to have limited the gap size, and thus the opening distance is unrelated to function. Fourth, a larger gap may simply be more efficient in trapping ssDNA.

### The 911 clamp binds other proteins besides the ATR kinase

The 9-1-1 clamp is reported to bind other proteins besides the ATR kinase. There is evidence that 911 binds and stimulates Pol β^59^. Pol β is mainly involved in base excision repair at a nick, or short gap. Considering the 9-1-1 ring would be loaded at a 5′ end, Pol β may interact with 9-1-1 ahead of it. The 9-1-1 clamp is also reported to bind the Fen1 nuclease^60^ and MYH (MutY glycosylase homolog)^61^. These factors are directly involved in repair of DNA damage, and thus the role of the 9-1-1 clamp may act directly in DNA repair, beyond its action as a trigger of the DNA damage response.

### Does 9-1-1 also load at 5′ Okazaki fragments?

The DNA damage response is not expected to be triggered by normal replication, yet the lagging strand is a rich source of 5′-recessed termini. Biochemical rapid reaction studies have shown that Okazaki fragments are processed and sealed exceedingly rapidly^62^. Hence, this rapid repair may ward off DNA damage sensing under normal conditions, but it is also possible that a process exists that prevents DNA damage factors from recognition of 5′ sites made during replication.

It is suggested that 9-1-1 might be involved in recombinational repair^11^. Indeed, there are several studies that invoke the role of Rad24 and 9-1-1 in various aspects of DNA recombinational repair, including double-strand-break repair, telomere maintenance and processes leading to cancer^63–68^.

In summary, the work described here provides the first structural support for a highly unusual and unexpected mechanism for the Rad24-RFC specificity for a 5′-recessed DNA junction. Our structures reveal that Rad24-RFC binds the dsDNA of a 5′-recessed DNA junction above the plane of an open ring, in which both the ring and the clamp loader are essentially in parallel with each other. Furthermore, the Rad24-RFC directs ssDNA into the 9-1-1 clamp, rather than dsDNA.

## Methods

### Cloning and expression of Rad24-RFC

The alternate clamp loader Rad24-RFC was expressed in *E. coli* by cloning the five genes into two compatible expression vectors that have different bacterial plasmid origins. The *rfc2*, *rfc3* and *rfc4* genes were cloned into pET3c (ColE1 origin) under control of the T7 promotor (ampicillin (Amp) selection). The *rad24* and *rfc5* genes were cloned into the pLANT vector (p15a origin), under control of the T7 promoter (kanamycin (Kan) selection). We have previously described the pLANT expression vector^69^. To express the Rad24-RFC, the pLANT-Rad24/Rfc5 and pET Rfc2, Rfc3, Rfc4 plasmids were co-transformed into competent *E. coli* BL21(DE3) CodonPlus cells that require Chloramphenicol (Cam) to maintain the CodonPlus plasmid that carries transfer RNAs common to eukaryotes but uncommon to *E. coli* (Invitrogen). The transformed cells were plated on LB medium containing 100 μg ml^−1^ Amp, 50 μg ml^−1^ Kan and 25 μg ml^−1^ Cam. A single colony was used to inoculate a 125-ml culture in LB medium containing these same antibiotics. After shaking at 37 °C for 3 h, 10 ml of cell culture was added to each of twelve 2-l fluted flasks, each containing 1 l of LB medium containing the same antibiotics. The flasks were incubated in a shaker-floor incubator for 19 h at 30 °C, at which time cultures reached an optical density at 600 nm (OD_600_) of 0.6. Cell cultures were then brought to 15 °C by swirling each flask in ice water, using a thermometer to gauge the temperature. Flasks were then placed in a different floor shaker that had been pre-equilibrated at 15 °C. Expression was then induced by adding 1 mM IPTG. Cells were allowed to express for 12 h, then collected by centrifugation and resuspended in 180 ml HEPES pH 7.5, 1 mM DTT, 0.5 mM EDTA, 20% glycerol and 400 mM NaCl.

### Purification of Rad24-RFC

Cells were lysed by a French Press using three passes at 22,000 psi for each pass. The lysate was clarified by centrifugation at 21,589*g* in a Sorvall SLA 1500 rotor for 1 h at 4 °C. Clarified lysate was diluted with buffer A (HEPES pH 7.5, 1 mM DTT, 0.5 mM EDTA and 20% glycerol) to a conductivity equal to 150 mM NaCl, then loaded onto a 125-ml SP Sepharose column equilibrated in buffer A containing 150 mM NaCl. The column was washed with buffer A + 150 mM NaCl until the UV absorption at 280 nm (UV_280_) reached a baseline level, then elution was performed with a 1-l linear gradient from 150 mM NaCl to 600 mM NaCl in buffer A. Fractions of 15 ml were collected and analyzed for Rad24-RFC by 10% sodium dodecyl sulfate polyacrylamide gel electrophoresis (SDS–PAGE). Fractions 16–22 (105 ml) were pooled. The pool was diluted 1.5-fold using buffer A to a conductivity equal to a solution of 200 mM NaCl, then loaded onto a 50-ml Fast Flow Q column pre-equilibrated in buffer A + 150 mM NaCl. The column was washed with buffer A + 150 mM NaCl until the UV_280_ approached a background value, then eluted with a 500 ml linear gradient of buffer A from 150 mM NaCl to 600 mM NaCl, collecting 7.5-ml fractions. Fractions 12–18 (120 mg) were pooled and aliquoted into 1-ml amounts and stored at −80 °C.

### Cloning and expression of the 9-1-1 complex

Rad17, Ddc1 and His-PK-Mec3 (Mec3 containing an N-terminal hexa-histidine tag, plus a seven-residue protein kinase A recognition site, LRRASLG)^70^ were cloned into pET21a for expression by T7 RNA polymerase. The pET-911 plasmid was transformed into BL21(DE3) CodonPlus cells (Invitrogen) and plated on LB medium plus 100 μg/ml of Amp and 25 μg/ml of Cam. A single colony was used to inoculate 125 ml LB medium containing 100 μg/ml of Amp and 25 μg/ml of Cam at 37 °C with shaking for 2 h, then 10 ml cell suspension was added to each of twelve 2-l fluted flasks that each contained 1 l LB medium containing 100 μg/ml of Amp and 25 μg/ml of Cam. The cell cultures were grown to an OD_600_ of 0.6, then each flask was swirled in ice water to bring the cultures to 15 °C. The flasks were transferred to a pre-cooled floor-shaker incubator at 15 °C and IPTG was added to 1 mM. Cells were induced for 10 h at 15 °C before collection by centrifugation, then the cells were resuspended in an equal volume (to the weight of the cell pellet—via weighing centrifuge bottles before and after cell collection) of 20 mM Tris-HCl pH 7.5, 10% wt/vol sucrose, and frozen at −80 °C.

### Purification of the 9-1-1 complex

Cell pellets equal to 6 l of cell expression culture were thawed and brought to a volume of 75 ml using buffer B (25 mM Tris-Cl pH 7.5, 1 mM DTT, 0.5 mM EDTA and 10% glycerol). The cells were then lysed by three passages through a French Press operating at 22,000 psi. The lysate was clarified by centrifugation at 21,589*g* in a Sorvall SLA 1500 rotor for 1 h at 4 °C. Clarified cell lysate (240 ml) was treated with 96 g (NH_4_)_2_SO_4_ with slow stirring for 30 min, then the protein precipitate was collected by centrifugation in an SLA rotor at 23,719*g* for 30 min at 4 °C. The supernatant was discarded and the pellet was resuspended in 24 ml of Ni-column binding buffer (20 mM Tris-Cl pH 7.9, 5 mM imidazole, 0.5 M NaCl, 10% glycerol) and dialyzed against 1 l of Ni column binding buffer overnight in the cold room. The protein was then loaded onto a 20-ml chelating Nickel HiTrap column (Sigma) that had been equilibrated in Ni-column binding buffer. The HiTrap column was washed with 40 ml of Ni column binding buffer + 60 mM imidazole, then eluted with a 200-ml linear gradient of 60 mM to 1 M imidazole in Ni-column binding buffer. Fractions of 2 ml were collected and then analyzed by SDS–PAGE. Fractions containing 9-1-1 were pooled, dialyzed against buffer B containing 150 mM NaCl overnight, and then loaded onto a 40-ml SP Sepharose column equilibrated in buffer B + 150 mM NaCl. The column was washed with 100 ml of buffer B + 150 mM NaCl, then eluted with a 400-ml linear gradient of 150 mM to 600 mM NaCl in buffer B. Fractions of 5 ml were collected and analyzed by SDS–PAGE. The 9-1-1 flowed through the column at this ionic strength, but many contaminants stuck to the column and were thus removed. The flowthrough, containing 9-1-1, was then dialyzed against Ni-column loading buffer and loaded onto a second Ni column with a bed volume of 5 ml. The loaded column was washed with 10 ml of Ni-column binding buffer + 60 mM imidazole, then eluted with a 50-ml gradient of 60 mM to 1 M imidazole in Ni-column binding buffer. Fractions of 1 ml were collected and analyzed by SDS–PAGE. Fractions containing 9-1-1 were pooled, and the nearly pure 9-1-1 was dialyzed against buffer B containing 50 mM NaCl. The protein was then loaded onto a 1-ml MonoQ column and eluted with a 10-ml linear gradient from 50 to 500 mM NaCl in buffer B. Fractions of 0.25 ml were collected and analyzed by SDS–PAGE. Fractions 29–33 contained pure 9-1-1 (~2 mg) and were aliquoted and stored at −80 °C.

### Cryo-electron microscopy grids preparation and data collection

The 3′-recessed DNA substrate was composed of the template strand (5′-CTG CAC GAA TTA AGC AAT TCG TAA TCA TGG TCA TAG CT-3′, 38 nt) and the primer strand (5′-AGC TAT GAC CAT GAT TAC GAA TTG-ddC-3′, 25 nt). DNA with both 3′- and 5′-recessed ends (double-tailed DNA) was composed of the upper shorter strand (5′-GCA GAC ACT ACG AGT ACA TA-3′, 20 nt) and the lower longer strand (5′-TTT TTT TTT TTA TGT ACT CGT AGT GTC TGC TTT TTT TTT T-3′, 40 nt). These DNAs were synthesized annealed, and then HPLC-purified by Integrated DNA Technologies. The assembly of yeast Rad24-RFC–9-1-1 clamp–DNA complex followed our previous procedure for assembling the *S.c.* Pol δ–PCNA–DNA complex in vitro^31^. Briefly, a droplet of 10 μl of purified 9-1-1 clamp at 3.3 μM and 0.4 μl of 3′-recessed DNA or the double-tailed DNA at 100 μM were mixed and incubated at 30 °C for 10 min, then the mixture and 0.75 μl 10 mM ATPγS were added into 3.2 μl of purified Rad24-RFC protein at a concentration of 8.6 μM. The final concentration of the reaction mixture was 1.8 μM Rad24-RFC, 2.2 μM 9-1-1 clamp, 2.7 μM DNA (either the 3′-recessed DNA or the double-tailed DNA), 0.5 mM ATPγS and 5 mM Mg acetate, with a total reaction volume of 15 μl. The mixture was then incubated in an ice-water bath for an additional 3 h (3′-recessed DNA) or 30 min (double-tailed DNA). The final molar ratio of Rad24-RFC:9-1-1 clamp:DNA was 1.0:1.2:1.5 with both DNA substrates. The Quantifoil Au R2/1 300 mesh grids were glow-discharged for 1 min in a Gatan Solarus, then 3 μl of the mixture was applied onto the EM grids. Sample vitrification was carried out in a Thermo Fisher Vitrobot Mark IV with the following settings: blot time 2 s, blot force 4, wait time 1 s, inner chamber temperature 6 °C and 95% relative humidity. The EM grids were flash-frozen in liquid ethane, cooled by liquid nitrogen. Cryo-EM data were automatically collected on a 300-kV Titian Krios electron microscope controlled by SerialEM in multi-hole mode. The micrographs were captured at a scope magnification of ×105,000, with the objective lens under-focus values ranging from 1.1 to 1.9 μm, by a K3 direct electron detector (Gatan) operated in super-resolution video mode. During a 1.5-s exposure time, a total of 75 frames were recorded with a total dose of 65 e^−^ Å^−2^. The calibrated physical pixel size was 0.828 Å for all digital micrographs.

### Image processing and 3D reconstruction

For the double-tailed DNA-bound ternary complex, data collection and image quality were monitored by cryoSPARC Live v3.2, installed in a local workstation^71^. The image preprocessing steps, including patch motion correction, contrast transfer function (CTF) estimation and correction, blob particle picking (70–150-Å diameter) and extraction with a binning factor of 2, were also carried out at the same time, and a total of 14,521 raw micrographs were recorded during a three-day real-time data-collecting session. About 8.5 million particles were extracted and subjected to two rounds of 2D image classification, resulting in ~471,000 ‘good’ particle images. These particles were used to calculate four starting 3D models. At this stage we observed two good 3D classes representing the Rad24-RFC–9-1-1 clamp–DNA complexes in clamp-closed and clamp-open conformations. Next we used Topaz (including training and picking) to pick more particles or particles in more views^72^. To avoid missing those less frequently occurring particle views, we used the recently reported ‘build and retrieve’ method to further clean up the particle images extracted by Topaz^73^. Finally, the cleaned-up particles were combined, and duplicate particles with 40% or larger particle diameter overlapping (56 Å) were removed. After homogeneous and non-uniform 3D refinement, we performed 3D viability analysis (3DVA) on three 3D EM modes in 30 iterations. This classified the particles into five 3D classes. A gate-closed conformation with ~147,000 particles and an open-gate conformation with ~300,000 particles were chosen for further non-uniform, per-particle CTF and local refinement, yielding the two final 3D maps at average resolutions of 3.17 Å (closed) and 3.23 Å (open), respectively. The 9-1-1 clamp regions in the two final 3D maps were more flexible and had weaker densities than the upper Rad24-RFC loader region. We performed 3D focused refinement and obtained the clamp densities to a resolution of 3.8 Å in the gate-closed state and to 4.3 Å resolution in the gate-open state, respectively. The improvement of the focused refinement was marginal. We thus used the two final full maps (without focused refinement) for model building.

For the sample mixed with the 3′-recessed DNA, we recorded 12,000 raw micrographs under similar data-collection procedures. After 2D image classification, about 230,000 particles were retained. Based on 2D and 3D classifications, we found that neither a binary complex of Rad24-RFC–9-1-1 clamp nor a ternary complex of Rad24-RFC–9-1-1 clamp–DNA formed in the presence of the 3′-recessed DNA.

### Model building, refinement and validation

For the atomic model building of the *S. cerevisiae* Rad24-RFC–9-1-1 clamp–DNA complex 3D maps in the closed and open states, we used the two homologous crystal structures of the human 9-1-1 clamp structure (PDB 3G65) and the yeast RFC–PCNA structure (PDB 1SXJ) as initial models for the yeast 9-1-1 and Rfc2–5, respectively. Because no Rad24 or its human homolog RAD17 structures were available, the de novo cryo-EM map modeling server DeepTracer (https://deeptracer.uw.edu) was used to produce the starting model^74^. The DNA density in our 3D EM map is sufficient to distinguish a purine from a pyrimidine. We used the de novo modeling program Map-to-Model wrapped in PHENIX^75^ to obtain the initial DNA model.

Although there are several human 9-1-1 clamp structures available^22–24^, model building for the yeast 9-1-1 ring was more challenging, because the map had lower resolution (3.8 Å) in this region (Extended Data Fig. 3), and the three subunits (Ddc1, 612 aa; Mec3, 474 aa; Rad17, 401 aa) are divergent from their respective human analogs (Rad9, 391 aa; Hus1, 280 aa; Rad1, 282 aa). There are several long insertions in each yeast subunit, in contrast to the structurally conserved PCNA-like core^18^. We thus adopted a homolog modeling approach using the human 9-1-1 clamp structure as a template. We first used the Phyre2 server (http://www.sbg.bio.ic.ac.uk/phyre2)^76^ to predict the structures of the three subunits. We next used I-TASSER^77^ and Robetta^78^ to predict these subunits independently. The models predicted by these three programs were very similar, and we chose the predictions from Phyre2 as the starting models. We only kept the PCNA-like core region and removed the peripheral insertion loops, because these loops are flexible and invisible in our EM maps. We then performed flexible fitting into the EM maps using the Flex-EM program wrapped in the CCP-EM package^79,80^.

Finally, the flexibly fitted 9-1-1 ring, the de novo built Rad24 and DNA models, and the yeast RFC–PCNA structure were fitted together into the EM map in the closed conformation. The PCNA and Rfc1 were then removed, and the remaining models were merged into a single file serving as the starting model of the complex in the UCSF Chimera^81^. This starting model was refined iteratively between the real space computational refinement in PHENIX and manual adjustment in COOT^82^. The final model of the closed conformation was refined to 3.2 Å and went through a comprehensive validation by the MolProbity program embedded in PHENIX^83^. The closed conformation model was used as the initial model for the open-state EM map. The model was iteratively refined and manually adjusted as described above, except for the 9-1-1 subunits Ddc1 and Mec3, which had much weaker EM densities; these two subunits were modeled by rigid body docking into the EM densities. Structure figures were prepared using ChimeraX^84^ and organized in Adobe Illustrator.

### Reporting Summary

Further information on research design is available in the Nature Research Reporting Summary linked to this Article.

## Online content

Any methods, additional references, Nature Research reporting summaries, source data, extended data, supplementary information, acknowledgements, peer review information; details of author contributions and competing interests; and statements of data and code availability are available at 10.1038/s41594-022-00742-6.

## Supplementary information

Reporting Summary

Supplementary Video 1 **Overall structures of the Rad24-RFC–9-1-1 clamp–DNA in the closed and open states**. The closed-state EM map is rotated around the *x* axis by 360º followed by another 360º rotation around the *y* axis. Next, the atomic model is shown morphing between the closed and the open states, highlighting the in-plane rotation of Mec3 that opens a 27-Å gap between Ddc1 and Mec3 of the 9-1-1 clamp. Note that the hook-like lower loop of Rad24 apparently controls the gate opening: the lower loop binds the Ddc1 subunit of the 9-1-1 camp in the closed state but becomes flexible and invisible in the open state.

Supplementary Video 2 **Specific interactions between Rad24-RFC and the 5′-recessed DNA junction**. DNA is in brown (upper shorter strand) and steel blue (lower longer strand), the Rad24 upper loop in magenta and lower loops in blue, the collar domain α-helix harboring the base mimicking residues in pale green, and the Rfc5 plug in cyan. First, the closed-state atomic model is rotated by 360º around the *x* axis and then around the *y* axis. Next, the scene transits to show the interaction between the tandem basic patch residues 81-HKRK-84 of Rad24 and the dsDNA region. This is followed by a scene showing the Rad24 residues at the 5′-recessed DNA junction. The final scene shows the Rad24 residues that interact and guide the 3′-overhang ssDNA in front of the 5′-recessed junction.

Supplementary Video 3 **Morph between Rfc1 of the** ***S.c.*** **RFC-PCNA structure and Rad24 of the S*.c.*** **Rad24-RFC–9-1-1 clamp–DNA structure**. Those two models are aligned by the loaders and shown as cartoons. First, the superimposed models are rotated around the *x* and *y* axes. RFC−PCNA is light blue with Rfc1 in salmon. Rad24-RFC–9-1-1 clamp–DNA is in rosy brown with Rad24 in pale green. Next is the morph between the two structures. During morphing, all regions are in transparent gray except for Rfc1 and Rad24. Note that Rad24 shifts 35 Å towards the upper right relative to Rfc1 to form a large groove for dsDNA binding. The final scene shows the Rad24 encircling the DNA.

## Data Availability

The 3D cryo-EM maps of *S. cerevisiae* Rad24-RFC–9-1-1 clamp–DNA complexes in the closed and open conformation at 3.2 Å resolution have been deposited in the Electron Microscopy Data Bank with accession codes EMD-25121 and EMD-25122. The corresponding atomic models have been deposited in the Protein Data Bank with accession codes 7SGZ and 7SH2. Source data are provided with this paper.

## Source data

Source Data Extended Data Fig. 1 Unprocessed SDS–PAGE gels.

## References

[1] Sancar A, Lindsey-Boltz LA, Unsal-Kacmaz K, Linn S (2004). Molecular mechanisms of mammalian DNA repair and the DNA damage checkpoints. Annu. Rev. Biochem..

[2] Harrison JC, Haber JE (2006). Surviving the breakup: the DNA damage checkpoint. Annu. Rev. Genet..

[3] Su TT (2006). Cellular responses to DNA damage: one signal, multiple choices. Annu. Rev. Genet..

[4] Zhou BB, Elledge SJ (2000). The DNA damage response: putting checkpoints in perspective. Nature.

[5] Abraham RT (2001). Cell cycle checkpoint signaling through the ATM and ATR kinases. Genes Dev..

[6] Walworth N, Davey S, Beach D (1993). Fission yeast *chkl* protein kinase links the *rad* checkpoint pathway to *cdc2*. Nature.

[7] Kumagai A, Guo Z, Emami KH, Wang SX, Dunphy WG (1998). The *Xenopus* Chk1 protein kinase mediates a caffeine-sensitive pathway of checkpoint control in cell-free extracts. J. Cell Biol..

[8] Peng CY (1997). Mitotic and G2 checkpoint control: regulation of 14-3-3 protein binding by phosphorylation of Cdc25C on serine-216. Science.

[9] Patil M, Pabla N, Dong Z (2013). Checkpoint kinase 1 in DNA damage response and cell cycle regulation. Cell. Mol. Life Sci..

[10] Donehower LA (2014). Phosphatases reverse p53-mediated cell cycle checkpoints. Proc. Natl Acad. Sci. USA.

[11] Ellison V, Stillman B (2003). Biochemical characterization of DNA damage checkpoint complexes: clamp loader and clamp complexes with specificity for 5′ recessed DNA. PLoS Biol..

[12] Majka J, Burgers PM (2003). Yeast Rad17/Mec3/Ddc1: a sliding clamp for the DNA damage checkpoint. Proc. Natl Acad. Sci. USA.

[13] Majka J, Binz SK, Wold MS, Burgers PM (2006). Replication protein A directs loading of the DNA damage checkpoint clamp to 5'-DNA junctions. J. Biol. Chem..

[14] Parrilla-Castellar ER, Arlander SJ, Karnitz L (2004). Dial 9-1-1 for DNA damage: the Rad9-Hus1-Rad1 (9-1-1) clamp complex. DNA Repair.

[15] Lieberman HB, Hopkins KM, Laverty M, Chu HM (1992). Molecular cloning and analysis of *Schizosaccharomyces pombe**rad9*, a gene involved in DNA repair and mutagenesis. Mol. Gen. Genet.

[16] Lieberman HB, Hopkins KM, Nass M, Demetrick D, Davey S (1996). A human homolog of the *Schizosaccharomyces pombe**rad9*^+^ checkpoint control gene. Proc. Natl Acad. Sci. USA.

[17] Hang H, Rauth SJ, Hopkins KM, Davey SK, Lieberman HB (1998). Molecular cloning and tissue-specific expression of Mrad9, a murine orthologue of the *Schizosaccharomyces pombe rad9*^+^ checkpoint control gene. J. Cell. Physiol..

[18] Venclovas C, Thelen MP (2000). Structure-based predictions of Rad1, Rad9, Hus1 and Rad17 participation in sliding clamp and clamp-loading complexes. Nucleic Acids Res..

[19] Zou L, Liu D, Elledge SJ (2003). Replication protein A-mediated recruitment and activation of Rad17 complexes. Proc. Natl Acad. Sci. USA.

[20] Majka J, Chung BY, Burgers PM (2004). Requirement for ATP by the DNA damage checkpoint clamp loader. J. Biol. Chem..

[21] Majka J, Niedziela-Majka A, Burgers PM (2006). The checkpoint clamp activates Mec1 kinase during initiation of the DNA damage checkpoint. Mol. Cell.

[22] Dore AS, Kilkenny ML, Rzechorzek NJ, Pearl LH (2009). Crystal structure of the rad9-rad1-hus1 DNA damage checkpoint complex—implications for clamp loading and regulation. Mol. Cell.

[23] Sohn SY, Cho Y (2009). Crystal structure of the human rad9-hus1-rad1 clamp. J. Mol. Biol..

[24] Xu M (2009). Structure and functional implications of the human rad9-hus1-rad1 cell cycle checkpoint complex. J. Biol. Chem..

[25] Delacroix S, Wagner JM, Kobayashi M, Yamamoto K, Karnitz LM (2007). The Rad9-Hus1-Rad1 (9-1-1) clamp activates checkpoint signaling via TopBP1. Genes Dev..

[26] Lee J, Kumagai A, Dunphy WG (2007). The Rad9-Hus1-Rad1 checkpoint clamp regulates interaction of TopBP1 with ATR. J. Biol. Chem..

[27] Enders GH (2008). Expanded roles for Chk1 in genome maintenance. J. Biol. Chem..

[28] Tannous EA, Yates LA, Zhang X, Burgers PM (2021). Mechanism of auto-inhibition and activation of Mec1(ATR) checkpoint kinase. Nat. Struct. Mol. Biol..

[29] Kelman Z (1997). PCNA: structure, functions and interactions. Oncogene.

[30] Bruck I, O’Donnell M (2001). The ring-type polymerase sliding clamp family. Genome Biol..

[31] Zheng F, Georgescu RE, Li H, O’Donnell ME (2020). Structure of eukaryotic DNA polymerase delta bound to the PCNA clamp while encircling DNA. Proc. Natl Acad. Sci. USA.

[32] Lancey C (2020). Structure of the processive human Pol delta holoenzyme. Nat. Commun..

[33] Madru C (2020). Structural basis for the increased processivity of D-family DNA polymerases in complex with PCNA. Nat. Commun..

[34] Kelch BA, Makino DL, O’Donnell M, Kuriyan J (2012). Clamp loader ATPases and the evolution of DNA replication machinery. BMC Biol..

[35] Kim J, MacNeill SA (2003). Genome stability: a new member of the RFC family. Curr. Biol..

[36] Erzberger JP, Berger JM (2006). Evolutionary relationships and structural mechanisms of AAA+ proteins. Annu. Rev. Biophys. Biomol. Struct..

[37] Bowman GD, O’Donnell M, Kuriyan J (2004). Structural analysis of a eukaryotic sliding DNA clamp–clamp loader complex. Nature.

[38] Gaubitz C (2020). Structure of the human clamp loader reveals an autoinhibited conformation of a substrate-bound AAA+ switch. Proc. Natl Acad. Sci. USA.

[39] Indiani C, O’Donnell M (2006). The replication clamp-loading machine at work in the three domains of life. Nat. Rev. Mol. Cell Biol..

[40] O’Donnell M, Kuriyan J (2006). Clamp loaders and replication initiation. Curr. Opin. Struct. Biol..

[41] Jeruzalmi D, O’Donnell M, Kuriyan J (2002). Clamp loaders and sliding clamps. Curr. Opin. Struct. Biol..

[42] Kelch BA (2016). Review: The Lord of the Rings: structure and mechanism of the sliding clamp loader. Biopolymers.

[43] Jeruzalmi D, O’Donnell M, Kuriyan J (2001). Crystal structure of the processivity clamp loader gamma (γ) complex of *E. coli* DNA polymerase III. Cell.

[44] Kelch BA, Makino DL, O’Donnell M, Kuriyan J (2011). How a DNA polymerase clamp loader opens a sliding clamp. Science.

[45] Miyata T (2005). Open clamp structure in the clamp-loading complex visualized by electron microscopic image analysis. Proc. Natl Acad. Sci. USA.

[46] Griffith JD, Lindsey-Boltz LA, Sancar A (2002). Structures of the human Rad17-replication factor C and checkpoint Rad 9-1-1 complexes visualized by glycerol spray/low voltage microscopy. J. Biol. Chem..

[47] Shiomi Y (2002). Clamp and clamp loader structures of the human checkpoint protein complexes, Rad9-1-1 and Rad17-RFC. Genes Cells.

[48] Bermudez VP (2003). Loading of the human 9-1-1 checkpoint complex onto DNA by the checkpoint clamp loader hRad17-replication factor C complex in vitro. Proc. Natl Acad. Sci. USA.

[49] Liu W (2019). The structure of the checkpoint clamp 9-1-1 complex and clamp loader Rad24-RFC in *Saccharomyces cerevisiae*. Biochem. Biophys. Res. Commun..

[50] Simonetta KR (2009). The mechanism of ATP-dependent primer-template recognition by a clamp loader complex. Cell.

[51] Cai J (1998). ATP hydrolysis catalyzed by human replication factor C requires participation of multiple subunits. Proc. Natl Acad. Sci. USA.

[52] Venclovas C, Colvin ME, Thelen MP (2002). Molecular modeling-based analysis of interactions in the RFC-dependent clamp-loading process. Protein Sci..

[53] Prestel A (2019). The PCNA interaction motifs revisited: thinking outside the PIP-box. Cell. Mol. Life Sci..

[54] Fukumoto Y, Ikeuchi M, Nakayama Y, Yamaguchi N (2016). The KYxxL motif in Rad17 protein is essential for the interaction with the 9-1-1 complex. Biochem. Biophys. Res. Commun..

[55] Piya G (2015). Characterization of the interaction between Rfa1 and Rad24 in *Saccharomyces cerevisiae*. PLoS One.

[56] Yuan Z, Li H (2020). Molecular mechanisms of eukaryotic origin initiation, replication fork progression, and chromatin maintenance. Biochem. J..

[57] Yuan Z (2017). Structural basis of Mcm2-7 replicative helicase loading by ORC-Cdc6 and Cdt1. Nat. Struct. Mol. Biol..

[58] Yuan Z (2020). Structural mechanism of helicase loading onto replication origin DNA by ORC-Cdc6. Proc. Natl Acad. Sci. USA.

[59] Toueille M (2004). The human Rad9/Rad1/Hus1 damage sensor clamp interacts with DNA polymerase β and increases its DNA substrate utilisation efficiency: implications for DNA repair. Nucleic Acids Res..

[60] Querol-Audi J (2012). Repair complexes of FEN1 endonuclease, DNA, and Rad9-Hus1-Rad1 are distinguished from their PCNA counterparts by functionally important stability. Proc. Natl Acad. Sci. USA.

[61] Shi G (2006). Physical and functional interactions between MutY glycosylase homologue (MYH) and checkpoint proteins Rad9-Rad1-Hus1. Biochem. J..

[62] Stodola JL, Burgers PM (2016). Resolving individual steps of Okazaki-fragment maturation at a millisecond timescale. Nat. Struct. Mol. Biol..

[63] Madabushi A, Lu A (2011). The novel role of cell cycle checkpoint clamp Rad9-Hus1-Rad1 (the 9-1-1 complex) in DNA repair. Adv. Med. Biol..

[64] Aylon Y, Kupiec M (2003). The checkpoint protein Rad24 of *Saccharomyces cerevisiae* is involved in processing double-strand break ends and in recombination partner choice. Mol. Cell. Biol..

[65] de la Torre-Ruiz M, Lowndes NF (2000). The *Saccharomyces cerevisiae* DNA damage checkpoint is required for efficient repair of double strand breaks by non-homologous end joining. FEBS Lett..

[66] Zubko MK, Guillard S, Lydall D (2004). Exo1 and Rad24 differentially regulate generation of ssDNA at telomeres of *Saccharomyces cerevisiae* cdc13-1 mutants. Genetics.

[67] Budzowska M (2004). Mutation of the mouse Rad17 gene leads to embryonic lethality and reveals a role in DNA damage-dependent recombination. EMBO J..

[68] Bao S (1999). HRad17, a human homologue of the *Schizosaccharomyces pombe checkpoint* gene *rad17*, is overexpressed in colon carcinoma. Cancer Res..

[69] Finkelstein J, Antony E, Hingorani MM, O’Donnell M (2003). Overproduction and analysis of eukaryotic multiprotein complexes in *Escherichia coli* using a dual-vector strategy. Anal. Biochem..

[70] Kelman Z, Naktinis V, O’Donnell M (1995). Radiolabeling of proteins for biochemical studies. Methods Enzymol..

[71] Punjani A, Rubinstein JL, Fleet DJ, Brubaker MA (2017). cryoSPARC: algorithms for rapid unsupervised cryo-EM structure determination. Nat. Methods.

[72] Bepler T (2019). Positive-unlabeled convolutional neural networks for particle picking in cryo-electron micrographs. Nat. Methods.

[73] Su CC (2021). A ‘build and retrieve’ methodology to simultaneously solve cryo-EM structures of membrane proteins. Nat. Methods.

[74] Pfab J, Phan NM, Si D (2021). DeepTracer for fast de novo cryo-EM protein structure modeling and special studies on CoV-related complexes. Proc. Natl Acad. Sci. USA.

[75] Adams PD (2010). PHENIX: a comprehensive Python-based system for macromolecular structure solution. Acta Crystallogr. D Biol. Crystallogr..

[76] Kelley LA, Mezulis S, Yates CM, Wass MN, Sternberg MJ (2015). The Phyre2 web portal for protein modeling, prediction and analysis. Nat. Protoc..

[77] Yang J (2015). The I-TASSER Suite: protein structure and function prediction. Nat. Methods.

[78] Song Y (2013). High-resolution comparative modeling with RosettaCM. Structure.

[79] Topf M (2008). Protein structure fitting and refinement guided by cryo-EM density. Structure.

[80] Burnley T, Palmer CM, Winn M (2017). Recent developments in the CCP-EM software suite. Acta Crystallogr. D Struct. Biol..

[81] Pettersen EF (2004). UCSF Chimera—a visualization system for exploratory research and analysis. J. Comput. Chem..

[82] Emsley P, Lohkamp B, Scott WG, Cowtan K (2010). Features and development of Coot. Acta Crystallogr. D Biol. Crystallogr..

[83] Chen VB (2010). MolProbity: all-atom structure validation for macromolecular crystallography. Acta Crystallogr. D Biol. Crystallogr..

[84] Pettersen EF (2021). UCSF ChimeraX: structure visualization for researchers, educators and developers. Protein Sci..

