# Supplementary material for: DNA is loaded through the 9-1-1 DNA checkpoint clamp in the opposite direction of the PCNA clamp
Source: Nat Struct Mol Biol. 2022 Mar 21;29(4):376–85. doi: 10.1038/s41594-022-00742-6 (PMC9010301; doi:10.1038/s41594-022-00742-6)
Supplement: Supplementary file 1 — Reporting Summary [file 41594_2022_742_MOESM1_ESM.pdf]

nature portfolio

Corresponding author(s): Huilin Li

Last updated by author(s): Dec 28, 2021

Reporting Summary

Nature Portfolio wishes to improve the reproducibility of the work that we publish. This form provides structure for consistency and transparency in reporting. For further information on Nature Portfolio policies, see our Editorial Policies and the Editorial Policy Checklist.

Statistics

For all statistical analyses, confirm that the following items are present in the figure legend, table legend, main text, or Methods section.

n/a	Confirmed	
☒	☐	The exact sample size (*n*) for each experimental group/condition, given as a discrete number and unit of measurement
☐	☒	A statement on whether measurements were taken from distinct samples or whether the same sample was measured repeatedly
☒	☐	The statistical test(s) used AND whether they are one- or two-sided<br>*Only common tests should be described solely by name; describe more complex techniques in the Methods section.*
☒	☐	A description of all covariates tested
☒	☐	A description of any assumptions or corrections, such as tests of normality and adjustment for multiple comparisons
☒	☐	A full description of the statistical parameters including central tendency (e.g. means) or other basic estimates (e.g. regression coefficient) AND variation (e.g. standard deviation) or associated estimates of uncertainty (e.g. confidence intervals)
☒	☐	For null hypothesis testing, the test statistic (e.g. *F*, *t*, *r*) with confidence intervals, effect sizes, degrees of freedom and *P* value noted<br>*Give P values as exact values whenever suitable.*
☒	☐	For Bayesian analysis, information on the choice of priors and Markov chain Monte Carlo settings
☒	☐	For hierarchical and complex designs, identification of the appropriate level for tests and full reporting of outcomes
☒	☐	Estimates of effect sizes (e.g. Cohen's *d*, Pearson's *r*), indicating how they were calculated

Our web collection on statistics for biologists contains articles on many of the points above.

Software and code

Policy information about availability of computer code

Data collection	SerialEM version 4.0.0 beta was used to collect the cryoEM micrographs.
Data analysis	cryoSPARC v3.2, Topaz, ChimeraX 1.2.5, Coot (version 0.9.5), Phenix (version 1.19.2-4158-000) and MolProbity (version 4.5).

For manuscripts utilizing custom algorithms or software that are central to the research but not yet described in published literature, software must be made available to editors and reviewers. We strongly encourage code deposition in a community repository (e.g. GitHub). See the Nature Portfolio guidelines for submitting code & software for further information.

Data

Policy information about availability of data

All manuscripts must include a data availability statement. This statement should provide the following information, where applicable:

- Accession codes, unique identifiers, or web links for publicly available datasets
- A description of any restrictions on data availability
- For clinical datasets or third party data, please ensure that the statement adheres to our policy

The accession PDB and EMDB codes of our two 3.2-Å resolution structures are shown as follows:
Rad24-RFC loader bound to the 9-1-1 clamp and DNA in a closed state, PDB: 7SGZ; EMDB: EMD-25121.
Rad24-RFC loader bound to the 9-1-1 clamp and DNA in an open state, PDB: 7SH2; EMDB: EMD-25122.

Field-specific reporting

Please select the one below that is the best fit for your research. If you are not sure, read the appropriate sections before making your selection.

☒ Life sciences ☐ Behavioural & social sciences ☐ Ecological, evolutionary & environmental sciences

For a reference copy of the document with all sections, see nature.com/documents/nr-reporting-summary-flat.pdf

Life sciences study design

All studies must disclose on these points even when the disclosure is negative.

Sample size	We collected a cryo-EM dataset with 14,521 raw micrographs.
Data exclusions	"Bad" raw particle images that did not produce 2D class averages or 3D class maps with defined features were excluded after 2D and 3D classifications. This criteria is empirical but is a standard image processing practice in the cryo-EM community.
Replication	Reproducibility resides in the large number of particles used to derive at the final 3D maps or 2D averages. The reliability and the resolution is measured by gold-standard Fourier shell correlation. Replication efforts with multiple refinement runs yielded was successful, yielding similar 3D maps.
Randomization	The raw particles were automatically selected by blob picking of the computer program (cryoSPARC v3.2).
Blinding	The investigators were not blinded to the specific data points during data collection and analysis, because visual inspection is necessary to ascertain the data quality.

Reporting for specific materials, systems and methods

We require information from authors about some types of materials, experimental systems and methods used in many studies. Here, indicate whether each material, system or method listed is relevant to your study. If you are not sure if a list item applies to your research, read the appropriate section before selecting a response.

Materials & experimental systems

n/a	Involved in the study
☒	☐ Antibodies
☒	☐ Eukaryotic cell lines
☒	☐ Palaeontology and archaeology
☒	☐ Animals and other organisms
☒	☐ Human research participants
☒	☐ Clinical data
☒	☐ Dual use research of concern

Methods

n/a	Involved in the study
☒	☐ ChIP-seq
☒	☐ Flow cytometry
☒	☐ MRI-based neuroimaging
