# Supplementary material for: DNA is loaded through the 9-1-1 DNA checkpoint clamp in the opposite direction of the PCNA clamp
Source: Nat Struct Mol Biol. 2022 Mar 21;29(4):376–85. doi: 10.1038/s41594-022-00742-6 (PMC9010301; doi:10.1038/s41594-022-00742-6)
Supplement: Source Data Extended Data Fig. 1 — Unprocessed SDS–PAGE gels. [file 41594_2022_742_MOESM5_ESM.pdf]

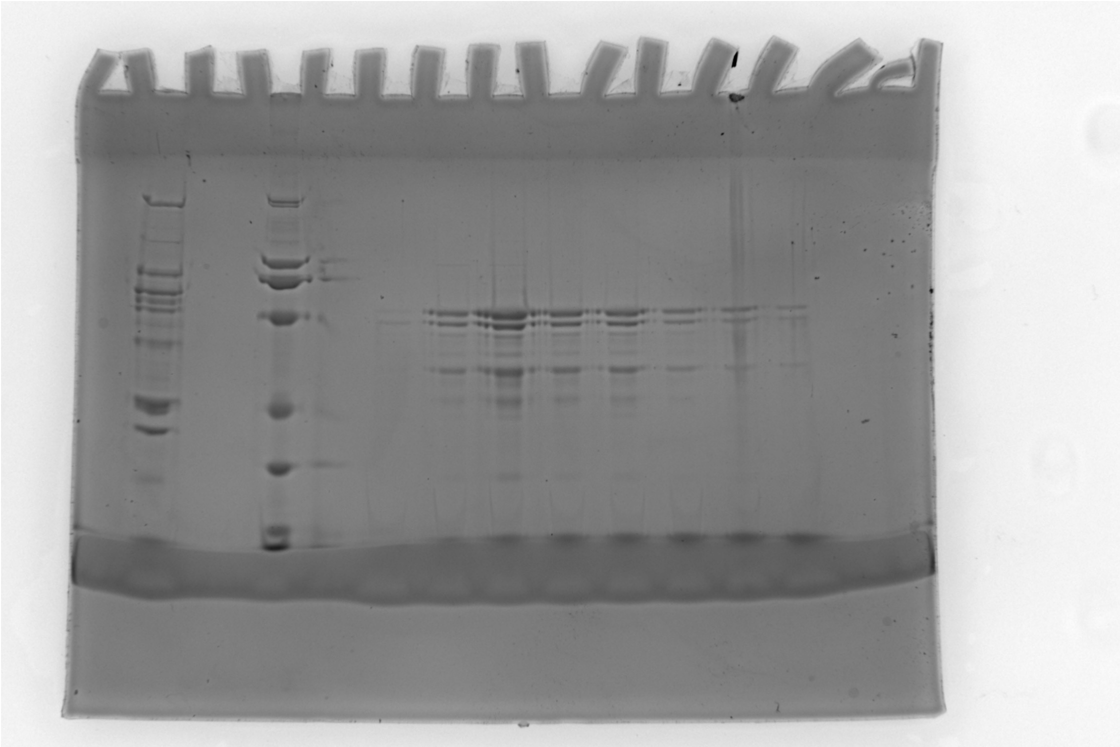

Raw gel image for Extended Data Figure 1a, left panel

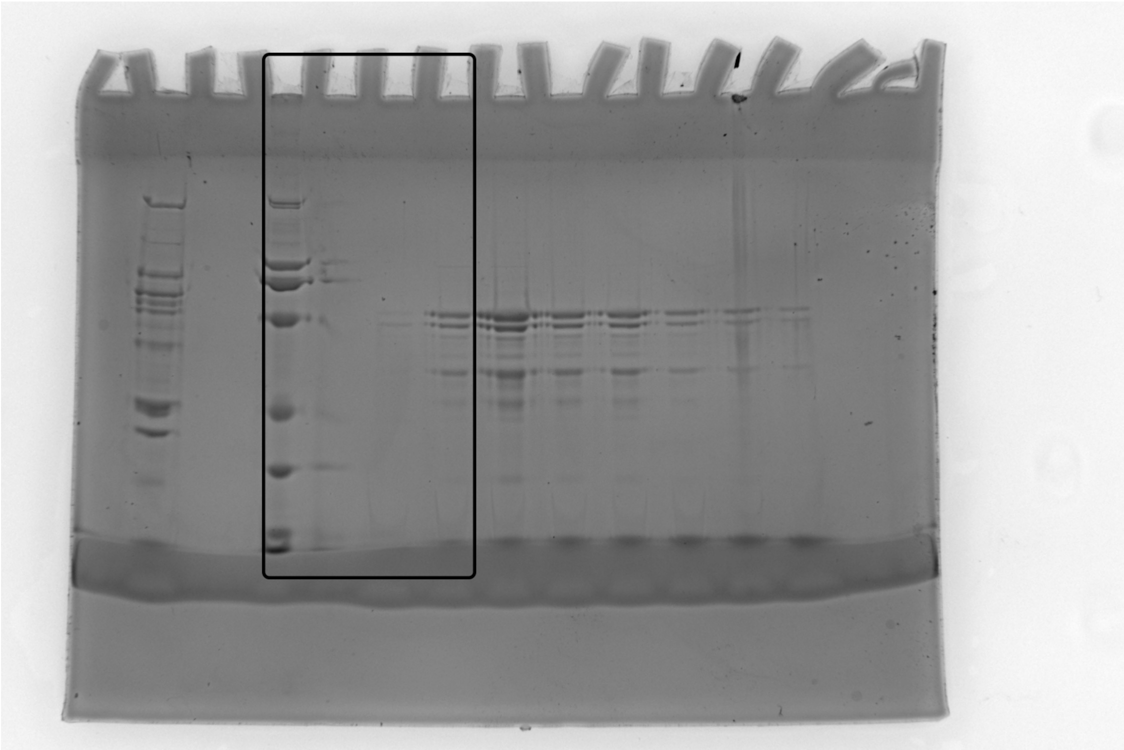

Area of the gel used in Extended Data Figure 1a left panel is boxed.

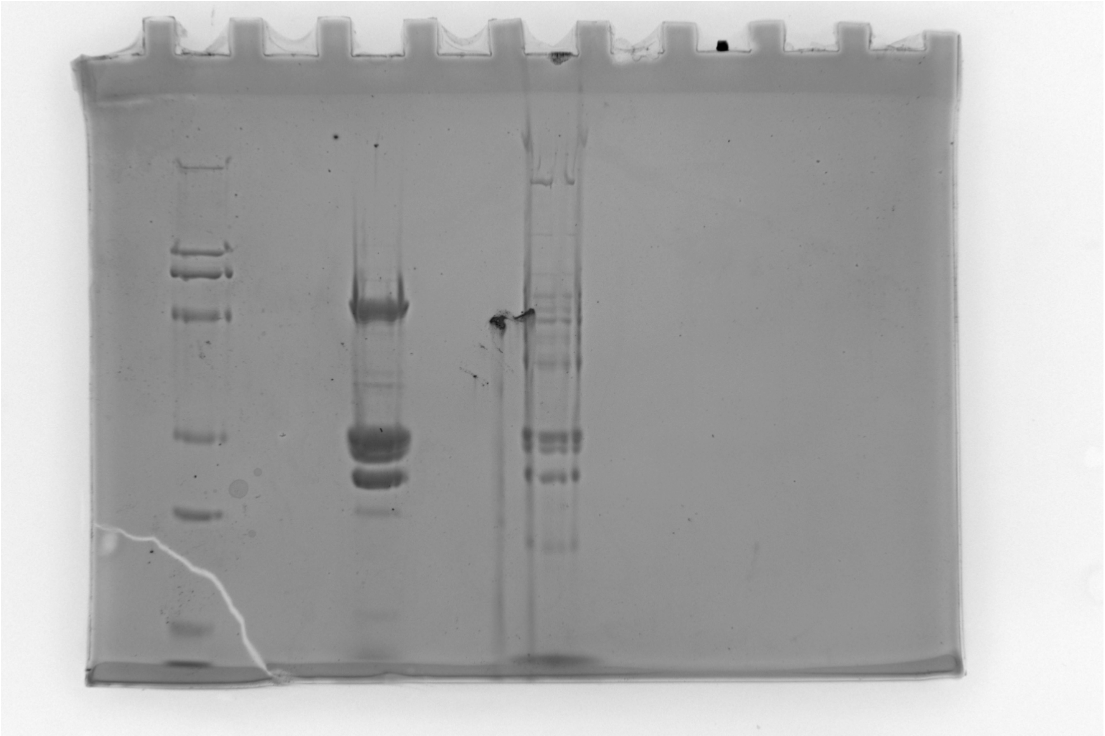

Raw gel image for Extended Data Figure 1a, right panel

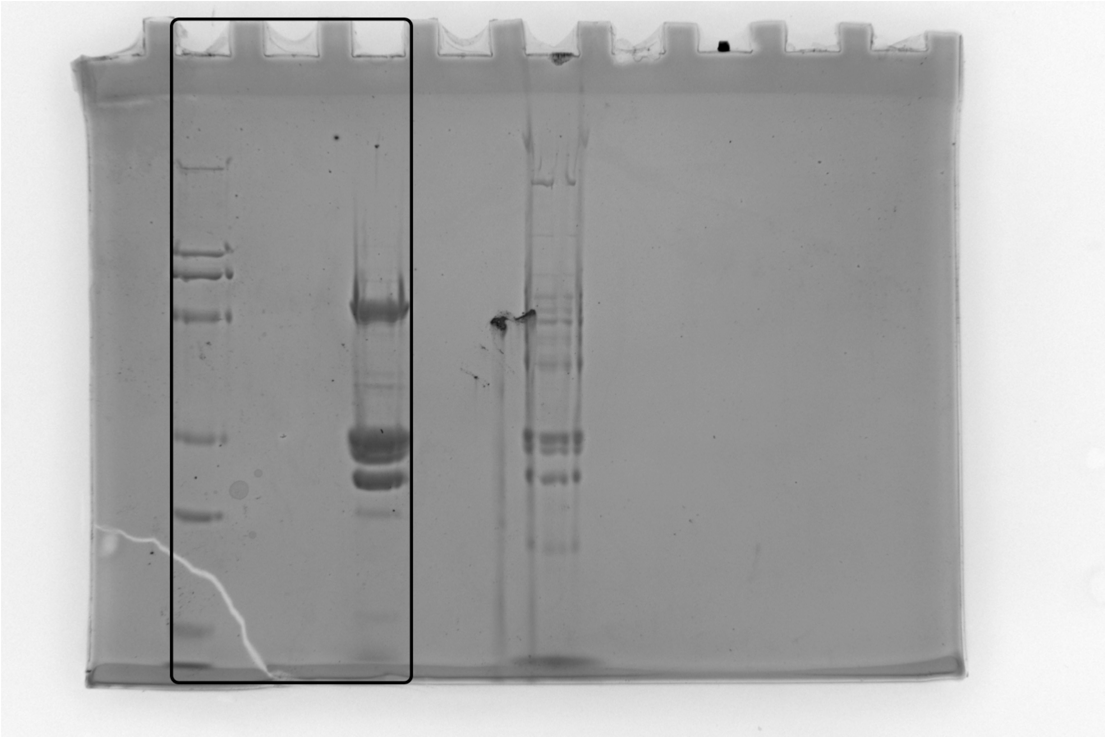

Area of the gel used in Extended Data Figure 1a right panel is boxed.
